# Development of Prolonged-Release Orodispersible Minitablets Containing Bisoprolol Fumarate

**DOI:** 10.3390/pharmaceutics18091080

**Published:** 2026-08-27

**Authors:** Justyna Srebro, Witold Brniak, Paulina Poloczek, Marian Paluch, Aleksander Mendyk

**Affiliations:** 1 Department of Pharmaceutical Technology and Biopharmaceutics, Faculty of Pharmacy, Jagiellonian University Medical College, Medyczna 9, 30-688 Cracow, Poland; justyna.srebro@doctoral.uj.edu.pl (J.S.); aleksander.mendyk@uj.edu.pl (A.M.); 2 Doctoral School of Medicinal and Health Sciences, Jagiellonian University Medical College, Św. Łazarza 16, 31-530 Cracow, Poland; 3 Institute of Physics, Faculty of Science and Technology, University of Silesia in Katowice, SMCEBI, 75 Pułku Piechoty 1a, 41-500 Chorzow, Poland

**Keywords:** prolonged release, microparticles, spray drying, ethylcellulose, orodispersible minitablets, pediatric dosage forms

## Abstract

**Background/Objectives**: Bisoprolol fumarate (BF) is a cardioselective beta-blocker used to treat pediatric heart failure and certain tachyarrhythmias. Currently, it is administered mainly as extemporaneously prepared suspensions from crushed tablets, which may cause issues with dose accuracy, stability, and palatability. Orodispersible minitablets (MODTs) may facilitate administration and individualized dosing, while prolonged release could potentially reduce peak-to-trough fluctuations and, thus, minimize adverse effects such as hypotension or bradycardia. This proof-of-concept study aimed to develop spray-dried prolonged-release microparticles containing BF, incorporate them into MODTs, and evaluate the effect of compression on drug release. **Methods**: Microparticles were prepared by spray drying ethanolic solutions of BF and ethylcellulose at 40 °C, 50 °C, and 60 °C. Their characterization included scanning electron microscopy, X-ray diffraction, differential scanning calorimetry, thermogravimetric analysis and dissolution studies. Selected microparticles were compressed into 3 mm MODTs, which were evaluated for mechanical properties, disintegration, and BF release. **Results**: Formulations containing 10% BF and 90% ethylcellulose released from 52.6% to 73% of BF after 2 h, increasing to 84.8–90.0% after 8 h and reaching 96% after 24 h. Solid-state analyses indicated complete amorphization of the drug in these microparticles. The spray-drying temperature affected process efficiency but did not significantly impacted morphology or dissolution behavior. The optimized MODTs disintegrated in less than 30 s and had a tensile strength of up to 2.62 MPa. Importantly, compression increased the initial BF release from 26.4% for the microparticles to 43.9% for the MODTs at 0.5 h, resulting in f_2_ values of 45.04–48.46. Despite the higher initial BF release, the moderately prolonged-release profile was maintained in the case of MODTs. **Conclusions**: These findings demonstrate the feasibility of combining prolonged-release microparticles with orodispersible minitablets as a proof-of-concept for a pediatric drug delivery approach.

## 1. Introduction

Sustained-release oral dosage forms are designed to provide prolonged drug release over an extended period of time. These formulations are particularly beneficial in the treatment of chronic diseases, as they reduce the frequency of dosing and, thus, improve patients’ compliance. Additionally, compared with immediate-release dosage forms, sustained-release formulations reduce fluctuations in plasma drug concentrations, contributing to improved drug safety, tolerability, and reduction in the severity of side effects [1,2].

One of the established approaches for the modification of drug release is the use of microparticles (MPs) as the functionalized drug carriers [3]. In addition to modulating drug release, microparticles can also be employed for taste and odor masking, protection of the active substance from environmental degradation, and improvement of drug stability and handling properties. Despite their small size, microparticles can be designed to provide the intended drug dissolution profile by adjusting their composition and morphology. Therefore, as a semi-finished product, they can be incorporated into various oral dosage forms, such as capsules, suspensions, or tablets (including minitablets and orodispersible tablets) [3,4].

MPs exhibit diverse morphologies and structures, including spheres, capsules, and liposomes, ranging usually from 1 to 1000 µm. Examples of polymers used to obtain sustained-release microparticles include pectin, pullulan, alginate, chitosan, ethylcellulose or other cellulose derivatives, methacrylate copolymers, poly(lactic acid) (PLA), and polylactic acid–glycolic acid copolymer (PLGA) [3,5]. Among the various methodologies employed for MP development, spray drying has emerged as a widely used and industrially scalable technique in pharmaceutical technology [6]. This process offers several advantages, including reproducibility, continuous operation, and the ability to encapsulate both hydrophilic and hydrophobic drugs within polymeric matrices [7,8].

Bisoprolol fumarate (BF), chemically present as the hemifumarate salt with a 2:1 bisoprolol-to-fumarate molar ratio, is a cardioselective β1-adrenergic receptor blocker used in the treatment of hypertension, angina pectoris, and chronic heart failure in adult patients [9,10,11,12]. Moreover, it is increasingly considered in children and adolescents with conditions such as dilated cardiomyopathy, congenital heart disease-associated heart failure, and certain tachyarrhythmias due to its moderate β1 selectivity, reducing the risk of bronchospasm compared with non-selective β-blockers [13,14]. In pediatrics, pharmacokinetic variability due to age-dependent maturation of hepatic metabolism and renal function necessitates careful dose titration and individualized therapy [15]. BF is characterized by high aqueous solubility and favorable pharmacokinetic properties, such as high bioavailability after oral administration, a long half-life (10–12 h), and relatively low hepatic first-pass metabolism [16]. However, conventional immediate-release formulations often require regular dosing to maintain therapeutic plasma concentrations [16]. A hypothetical advantage of developing a prolonged-release BF formulation may be a reduction in fluctuations in plasma drug concentrations, which could potentially result in more consistent pharmacodynamic effects. However, whether this would translate into improved tolerability or a lower incidence of adverse effects, such as bradycardia, hypotension, dizziness, and fatigue, also remains to be established due to the relatively long half-life of BF. Therefore, the clinical relevance and potential therapeutic benefits of prolonged-release BF formulations would require confirmation in appropriate pharmacokinetic and pharmacodynamic studies. Another potential clinical benefit of developing such a drug formulation—which would require experimental confirmation—could be related to potential taste masking. The incorporation of BF into sustained-release microparticles may be beneficial, as it could reduce the amount of drug released in the oral cavity during administration.

Despite these advantages, achieving prolonged release of BF is challenging because its high solubility in water can promote rapid dissolution and diffusion through the polymeric matrix, potentially resulting in an initial burst release [17]. Effective modification of drug release requires the selection of appropriate polymeric matrices capable of limiting water penetration and drug diffusion. In this context, spray drying represents an enabling technology for the encapsulation of bisoprolol fumarate into polymer-based microparticles designed to provide sustained drug release.

To date, several studies have reported on the development and characterization of micro- and nanoparticulate delivery systems with bisoprolol fumarate. One of these studies proposed the formulation of solid lipid microparticles via a melt emulsification technique. The particulate matrix, composed of carnauba wax and ceresin wax, was designed to achieve controlled release of bisoprolol fumarate [18]. Sharma et al. [19] reported the preparation of nanoparticles via ionotropic gelation of an aqueous aloe vera gel solution incorporating BF, atenolol, or propranolol. The resulting formulations demonstrated sustained-release characteristics, with the BF-loaded nanoparticles exhibiting the highest release of the active pharmaceutical ingredient, reaching approximately 85% [19]. The application of poly(lactic-co-glycolic acid) (PLGA) nanoparticles encapsulating bisoprolol fumarate for intraocular delivery in the management of glaucoma has also been proposed. A prolonged release of BF over 12 h was obtained [20]. Microparticles containing other APIs belonging to the class of β-adrenergic receptor antagonists have also been developed [21,22]. Stojmenovski et al. [22] formulated sodium alginate-based microparticles encapsulating propranolol hydrochloride, which exhibited controlled-release characteristics [22].

Another study reported the formulation of a hydrogel matrix composed of polyvinyl alcohol and chitosan, which prolonged the release of BF up to 72 h. Furthermore, the freeze-drying process was observed to decrease the cumulative amount of BF released throughout the dissolution study [23]. The formulation of sustained-release tablets [24] and minitablets [25] containing bisoprolol fumarate has also been reported, employing a hydrophilic polymer-based matrix system composed specifically of hydroxypropyl methylcellulose and methylcellulose. Moreover, orodispersible films [26] and immediate-release tablets based on electrospun fibers containing BF have also been developed [27]. In contrast, Ibrahim [28] formulated orodispersible tablets containing BF, employing crospovidone as a superdisintegrant and Eudragit E100 as a taste-masking polymer. Furthermore, sublingual tablets were developed, and the optimized formulation exhibited a disintegration time of approximately 35 s [28].

In addition to conventional oral dosage forms, a promising direction for the development of modified-release formulations of bisoprolol fumarate is provided by transdermal delivery systems such as patches [29], gels [30,31], and films [32]. A representative example is the Bisono^®^ controlled-release transdermal patch, which has been introduced to the Japanese market for the management of hypertension and tachycardia associated with atrial fibrillation [33].

Currently, BF for pediatric use is typically administered as an extemporaneously prepared suspension obtained from crushed tablets, which may be associated with issues related to dose accuracy, stability, and palatability [34]. Given these limitations, the development of solid dosage forms dedicated to pediatric patients is justified. One of the promising alternatives for children addressing common challenges such as swallowing difficulties, poor compliance, and the need for flexible dosing is the use of orodispersible dosage forms, especially orodispersible minitablets (MODTs) [35]. They rapidly disintegrate in the oral cavity without the need for water and can be administered even for neonates [36,37,38]. They improve convenience and acceptability and enable precise and individualized dose titration through the administration of multiple small units, which is especially advantageous in weight-based dosing [39]. Their small size facilitates easy swallowing while maintaining dose accuracy and minimizing the risk of choking, thereby supporting improved adherence and therapeutic outcomes. MODTs may be administered as a single or multiple units to provide an accurate individualized dose either directly into the oral cavity or using a spoon. As they quickly disintegrate upon contact with a small volume of liquid, they may also be convenient for mixing with thickened liquids, pudding, sauces, milk, infant formulas, or water. In the case of patients with enteral feeding tubes, the MODTs may be easily dispersed directly in an enteral syringe and easily administered in a suspension form [35,40,41]. To date, no MODTs containing bisoprolol fumarate have been commercially developed or reported in the literature.

Therefore, the aim of this study was to develop and characterize spray-dried prolonged-release microparticles containing bisoprolol fumarate and to incorporate them into orodispersible minitablets, as a proof of concept, with particular emphasis on evaluating the impact of compression on the release of the drug substance.

## 2. Materials and Methods

### 2.1. Materials

Bisoprolol fumarate (BF, CAS: 104344-23-2; IUPAC name: (E)-but-2-enedioic acid;methane;1-(propan-2-ylamino)-3-[4-(2-propan-2-yloxyethoxymethyl)phenoxy]propan-2-ol) was purchased from Wuhan ChemNorm Biotech Co. Ltd., Wuhan, China. Ethocel 20 Standard Premium (medium-molecular-weight ethylcellulose) was kindly gifted by Colorcon Ltd., Budapest, Hungary. For the preparation of the solutions, 96% ethanol (*v*/*v*) or water produced by an Elix 15UV Essential Reverse Osmosis System (Merck KGaA, Darmstadt, Germany) was used.

For the preparation of the tablet blends, microcrystalline cellulose (Vivapur 101, JRS Pharma GmbH, Rosenberg, Germany) was used as a diluent, disintegrant and dry binder; croscarmellose sodium (Primellose, DFE Pharma, Goch, Germany) and sodium starch glycolate (Primojel, DFE Pharma, Goch, Germany) served as superdisintegrants; fumed silica (Aerosil 200, Evonik Operations GmbH, Darmstadt, Germany) was used as a glidant to improve powder flow; and sodium stearyl fumarate (Lubripharm SSF, SPI Pharma, Wilmington, DE, USA) served as a lubricant.

### 2.2. Methods

#### 2.2.1. Preparation of Microparticles

Ethylcellulose was selected as the prolonged-release polymer based on the preliminary spray-drying studies. Several polymers commonly used in modified-release formulations were evaluated, including EC, shellac, Eudragit RS PO, and sodium alginate. Among them, EC provided the most promising overall performance (sufficient yield of the spray-drying process, formation of a fine powder product, and retardation of BF release) and was, therefore, selected for the further formulation studies. Its choice was also supported by its good solubility in ethanol, non-ionic and chemically inert character, pH-independent properties, and reported high plasticity. However, EC was selected based on its experimentally observed performance in our formulation rather than because it is superior to other prolonged-release polymers. In the preliminary experiments, ethylcellulose microparticles were prepared at 50 °C; therefore, this temperature was selected as a starting point for the described studies.

A systematic experimental matrix comprising 15 different microparticle formulations was investigated. The formulations represented all combinations of two factors: (i) the BF-to-ethylcellulose mass ratio, assessed at five levels (1:9, 1:3, 1:1, 3:1, and 9:1), and (ii) the processing temperature at three levels (40 °C, 50 °C, and 60 °C). Each combination was represented by one independently prepared spray-dried batch. In order to produce microparticles, ethanolic solutions of ethylcellulose were prepared at concentrations ranging from 1% to 9%, depending on the formulation, and BF was subsequently dissolved in these solutions. The resulting solutions were immediately spray-dried using a Büchi B-190 Mini Spray-Dryer (Büchi Labortechnik AG, Flawil, Switzerland). The spray nozzle diameter was 0.7 mm, the atomizing air flow was 600 NL/min, and the pump efficiency was set to 6% (corresponding to a feed rate of approx. 1.22–1.66 mL/min, depending on the solution). After the process was completed, the resulting microparticles were further dried for 20 min at the same temperature and air flow rate as during the process. The composition of the 15 prepared formulations and the process temperatures used for their preparation are presented in Table 1.

To ensure systematic traceability throughout the experimental stage, a standardized nomenclature was implemented. Microparticles were labeled BF_EC_X:Y_Z, where BF and EC are the bisoprolol fumarate and ethylcellulose; X:Y is the drug-to-polymer mass ratio (from 1:9 to 9:1); and Z is the spray-drying temperature (40 °C, 50 °C, or 60 °C). To maintain nomenclature clarity in specific sections of this manuscript, the omission of the temperature suffix implies a general discussion focusing solely on the ingredient mass ratio, thereby excluding the process temperature. For the subsequent tableting stage, only microparticles spray-dried at 60 °C were used; therefore, the resulting minitablets were labeled MT_BF_EC_X:Y_C, omitting the temperature value. MT indicates minitablets, and C is the compaction force in Newtons (N).

#### 2.2.2. Evaluation of Microparticles

##### Morphological Assessment

The surface characteristics of the microparticles were assessed using scanning electron microscopy (SEM). The samples were sputtered with gold and examined using a Hitachi S-4700 scanning electron microscope (Hitachi High-Tech, Tokyo, Japan). SEM imaging was carried out at magnifications of 50×, 100×, 250×, 500×, 1000×, and 2000×, with an acceleration voltage of 20 kV.

##### DSC and XRPD Studies

The thermal behavior of raw API, polymer, their physical mixture (1:1), and microparticles was investigated using a Netzsch Differential Scanning Calorimeter 204 F1 Phoenix (Selb, Germany). The weighed samples were heated in a nitrogen atmosphere (20 mL/min) in the temperature range of 20 °C to 250 °C in standard pans with a pierced lid. Due to the thermal decomposition of BF observed at temperatures above approximately 180 °C (Figure S1), the samples were measured in a single heating cycle. A second heating cycle was not performed, as heating above 200 °C, required for complete melting of the ethylcellulose component, could thermally alter the sample through BF decomposition and formation of degradation products. The heating rate used for all measurements was 10 °C/min.

The prepared samples were also analyzed by powder X-ray diffraction (XRPD). The study was performed using a Rigaku MiniFlex 600 X-ray diffractometer (Tokyo, Japan). The diffraction patterns were collected in a 2θ range between 3° and 40°, with a step of 10°/min. Measurements were carried out once per sample without replication.

##### Particle Size Determination

Particle size distribution was determined using a Mastersizer 3000 laser diffraction particle size analyzer (Malvern Instruments Ltd., Worcestershire, UK) equipped with a Hydro EV semi-automated wet dispersion unit and a Hydro Sight dynamic imaging module. The dispersant used during the analysis was purified water obtained from a reverse osmosis system, Elix Essential 15 UV (Merck Millipore, Molsheim, France), maintained at ambient temperature (approximately 20–25 °C) and stirred continuously at 1500 rpm throughout the measurement.

Prior to analysis, the instrument performed automatic laser alignment, and the background signal was recorded using the Mastersizer 3000 software (version 3.60). The powdered samples were initially dispersed manually in a test tube in the 0.01% sodium lauryl sulfate solution to enhance particle wetting and prevent flotation. Immediately after dispersion, the samples were gradually introduced into the measurement cell until an obscuration level between 5% and 20% was reached. Measurements were conducted over a particle size range of 0.01–1000 µm. Each sample was analyzed in six independent replicates.

The particle size distribution was calculated using the Fraunhofer diffraction model, based on the angular distribution of scattered light intensity. The Mastersizer software was subsequently used to calculate mean values from the six measurements and to determine the characteristic particle size parameters D10, D50, and D90 (10th percentile particle diameter, median particle diameter, and 90th percentile particle diameter, respectively). The Span value, describing the width of the particle size distribution, was calculated using the OriginPro 2024b software according to the following equation:

$$Span=\frac{D_{90}-D_{10}}{D_{50}}$$

Microparticles containing 90% BF exhibited very high solubility in water, which prevented reliable particle size determination in water. Consequently, these samples were excluded from particle size analysis.

##### Determination of Drug Loading

Microparticle samples corresponding to a theoretical BF content of 7.5 mg were accurately weighed into 100 mL volumetric flasks. Subsequently, 50 mL of purified water was added, and the samples were shaken at 320 rpm for 24 h using a laboratory shaker (Heidolph Unimax 1010, Schwabach, Germany).

The suspensions were filtered through the nylon syringe filters (0.45 µm), and the BF content was determined using a UV-VIS spectrophotometer (Shimadzu UV-1900, Suzhou, China) equipped with a 10 mm quartz cuvette. The detection of the sample was set at λ = 271 nm. The calibration curve for BF was linear in the concentration range of 0.2 to 200.0 μg/mL (R^2^ = 0.9999). Linearity was evaluated using 9 different concentration levels pre-prepared from five independently weighed and dissolved BF samples. Each tested formulation was analyzed in six replicates.

The actual drug loading was calculated based on the concentration measurements according to the following formula:

Actual drug loading%ww=mass of BF measured in the samplemass of microparticles used for analysis ×100

Mean values and standard deviations were calculated for six independent samples.

The drug-loading efficiency was calculated based on the following formula:

$$Drug\text{-}loading\;efficiency\left[\%\right]=\frac{actual\;drug\;loading}{theoretical\;drug\;loading}\;\times 100$$

The process yield was calculated as the mass of dry powder recovered from the collecting chamber and cyclone divided by the total mass of BF and EC used to prepare the spray-drying solution.

Drug recovery was calculated according to the following formula:

$$Drug\;recovery=\;\frac{process\;yield\times drug-loading\;efficiency\;}{100}$$

##### Dissolution Studies

In vitro dissolution studies of the microparticles were performed using a Ph. Eur. 11.0 type 2 paddle apparatus (Hanson Research SR8 Plus Dissolution Test Station, Chatsworth, CA, USA). The dissolution medium consisted of 500 mL of purified water, kept at 37 ± 0.5 °C, with a paddle rotation speed of 75 rpm. The dissolution medium and parameters were selected according to the USP monograph for bisoprolol fumarate tablets. The samples were collected at 30 min, 1, 2, 4, 8, 12, and 24 h of the study. The sample volume was automatically refilled with the same amount of medium. Dissolution testing was conducted in triplicate for each formulation. For each microparticle formulation, three individual samples were precisely weighed. Each sample corresponded to a theoretical BF content of 20 mg.

To prevent the flotation of the microparticles on the surface of the dissolution medium, they were introduced into the dissolution vessels as an extemporaneously prepared suspension. For sample preparation, an amount of microparticles corresponding to the required dose was accurately weighed into 25 mL volumetric flasks. Subsequently, 10 mL of purified water was added using an automatic pipette, and the samples were shaken at 320 rpm for 2 min using a laboratory shaker to ensure uniform dispersion. The resulting suspensions were then quantitatively transferred into the dissolution vessels. Each flask was subsequently rinsed with an additional 10 mL of purified water to ensure complete transfer of the microparticles, and the rinsing medium was also transferred to the corresponding vessel. BF release was monitored by automatic UV spectrophotometric analysis using a Shimadzu UV-1800 spectrophotometer (Shimadzu, Suzhou, China) at λ = 271 nm.

#### 2.2.3. Compression of Microparticles to Obtain Minitablets

The selected microparticle formulations were mixed with excipients and compressed into minitablets with a diameter of 3 mm. Three types of minitablet blends were prepared, according to the composition presented in Table 2. Each minitablet blend contained a different type of MPs, namely, BF_EC_9:1_60, BF_EC_1:1_60, or BF_EC_1:9_60. For each minitablet, the amounts of MPs and excipients were calculated to contain 1 mg of BF and a minitablet weight of 16 mg. The minitablets formulated with BF_EC_1:9 microparticles were characterized by the highest proportion of microparticle content and the lowest proportion of microcrystalline cellulose, namely, 24% and 62.5%. To ensure an equivalent dose of BF in other tablet blends, the fraction of microcrystalline cellulose was increased proportionally to the reduction in microparticle content. The minitablets were compressed with compression forces of 150, 250, 350, and 400 N, which correspond to compression pressure values of 21.2, 35.4, 49.5 and 56.6 MPa, respectively.

Compression was performed using an EZ-SX texture analyzer (Shimadzu, Kyoto, Japan) equipped with a specially designed compression probe adapted from an EK0 eccentric tablet press. The compression assembly consisted of a single punch with a diameter of 3 mm attached to the analyzer’s load cell and a die mounted on the flat surface of the instrument table. The maximum testing capacity of the load cell was 500 N.

#### 2.2.4. Evaluation of Minitablets

##### Morphology

The morphological characteristics of the selected series of minitablets were evaluated by scanning electron microscopy using the same method as described previously for the microparticles. SEM images were obtained for both the outer surface of the minitablets and the internal structure, following manual splitting of the tablets into approximately equal halves.

##### Tensile Strength

The conventional tablet hardness tester available in our laboratory was not suitable for the measurement of resistance to crushing of minitablets, as its lower measuring range starts at 0.4 kp (3.9 N), which was very close to the lowest crushing force values registered for the prepared minitablets. In several cases, the breaking point was not even detected by the pharmacopeial hardness tester because minitablets were partially pushed beneath the instrument jaws. Therefore, the resistance to crushing of the minitablets was determined using an EZ-SX texture analyzer (Shimadzu, Suzhou, China) equipped with a flat cylindrical probe (10 mm in diameter) and a 500 N load cell with a measuring range starting at 1 N and a force accuracy of 0.5%. During measurement, the probe moved at a constant speed of 10 mm/min toward the minitablet placed vertically on the instrument table. The resistance to crushing of minitablets was registered as the maximum force required to break each minitablet. Measurements were conducted on six tablets per batch. The tensile strength was calculated according to the following equation:

$$T_{s}=\;\frac{2F}{\pi Dh}$$

where *F* is the resistance to crushing of minitablets [N], *D* is the minitablet diameter [mm], and *H* is the minitablet thickness [mm].

The results were expressed as mean values with corresponding standard deviations.

##### Disintegration Time

The disintegration time of the minitablets from each batch was determined using the BJKSN-13 apparatus, developed at the Department of Pharmaceutical Technology and Biopharmaceutics of the Jagiellonian University Medical College and described previously in several works [25,42,43]. Briefly, the apparatus consisted of an external chamber filled with 5 mL of water and an internal chamber in which a tablet was fixed to a rotating shaft and brought into contact with a small volume of water (~0.5 mL). During the test, the shaft performed a half-rotation movement and a mechanical stress, simulating the motions of the tongue in an oral cavity. A magnetic sensor system enables continuous measurement of changes in tablet thickness over time, allowing the determination of disintegration time. The test was conducted on six minitablets from each formulation. The results were expressed as mean values with corresponding standard deviations for each batch of minitablets tested.

##### Dissolution Studies

Dissolution studies for minitablets were conducted under the same conditions as those for microparticles. Six minitablets were placed in a dissolution apparatus vessel filled with 500 mL of water. The samples were collected at 30 min, 1, 2, 4, 8, 12, and 24 h of the study. A dissolution study was conducted in triplicate for each formulation. Each investigated sample of minitablets corresponded to the theoretical content of 6 mg of BF.

#### 2.2.5. Analysis of Release Kinetics

An analysis of drug release kinetics was performed using the open-source RKinetDS software (ver. 3.0) [44], which enables fitting experimental dissolution data to established mechanistic and empirical models. The calculations were carried out for microparticles exhibiting prolonged drug release (BF_EC_1:9_40, BF_EC_1:9_50, and BF_EC_1:9_60), as well as for minitablets from the MT_BF_EC_1:9_60 batches. Other microparticle and minitablet formulations were excluded from the analysis due to their immediate-release dissolution profile. Models describing API release from a polymer matrix without a lag time in drug diffusion (Korsmeyer-Peppas [45] and Peppas-Sahlin [46]) or an erosion mechanism (Hixson-Crowell [47] and Hopfenberg [48]) were selected for analysis [49,50]. The fitting of the model was performed using a minimum of 3 experimental data points, excluding 0 and data points corresponding to drug release that exceeded 65%. The model’s predictability was assessed based on the root-mean-square error (RMSE) values.

#### 2.2.6. Statistical Analysis

For statistical comparison of the obtained dissolution profiles, the mean dissolution time (MDT) and the area under the curve (AUC) were calculated. The MDT was determined using the equation proposed by Podczeck et al. [51]. The AUC values were calculated using the trapezoidal method over the time interval from 0 to 24 h. The calculated AUC values were normalized to the actual BF doses contained in the samples of microparticles and minitablets, to enable a direct comparison of the resulting data. Dissolution profile similarity was additionally evaluated using the model-independent similarity factor f_2_ for minitablets with prolonged-release characteristics. The dissolution profile of the bulk microparticles incorporated into the minitablets was used as the reference and compared with the profiles of minitablets MT_BF_EC_1:9 prepared at different compression forces. Pairwise f_2_ comparisons were also performed between minitablets of the same formulation, MT_BF_EC_1:9, compressed at different forces to evaluate the effect of compression force on drug release. The f2 was calculated according to the following equation:

$$f_{2}=50\cdot log\left[\frac{100}{\sqrt{1+\;\frac{\sum_{t=1}^{t=n}{\left[R_{t}-T_{t}\right]}^{2}}{n}}}\right]$$

where *n* is the number of sampling time points, *R_t_* is the mean cumulative percentage of BF released from the reference formulation at time t, and *T_t_* is the mean cumulative percentage of BF released from the test formulation at time t.

An *f*_2_ value between 50 and 100 was considered to show profile similarity in accordance with regulatory guidance [52]. The values of f_2_ were not calculated for other minitablets due to their fast dissolution properties. The amount of BF released from microparticles at the first time point exceeded 85%.

Data analysis was performed using the OriginPro 2024b software (version 10.1.5.132) and Microsoft Excel (Microsoft 365). The statistically significant differences between formulations were assessed using one-way analysis of variance (ANOVA), followed by Tukey’s multiple-comparison post hoc test. A *p*-value of less than 0.05 was considered statistically significant (Tables S2–S10).

## 3. Results

### 3.1. Spray-Drying Process

During the spray-drying process, differences in process efficiency were observed for formulations processed with different inlet air temperatures. The lowest process efficiency was noted for microparticles composed of 10% BF and 90% EC. For microparticles dried at 40 °C, the yield was only 22.9%, increasing to 36.7% at 50 °C and 49.5% at 60 °C (Table S1). The pronounced product losses were primarily attributed to particle deposition on the walls of the drying chamber. Furthermore, the collected material exhibited a high electrostatic charge and a pronounced tendency to agglomerate.

An increase in API content resulted in enhanced process efficiency across the entire investigated temperature range. The highest yield (66.8%) was achieved for microparticles containing 75% BF and 25% EC when dried at 60 °C. The obtained powder was characterized by poor flowability, although no evident agglomeration was observed. A further increase in BF content to 90% led to a similar process efficiency, with yields of 51.8% and 65.5% for microparticles dried at 40 °C and 60 °C, respectively. For these formulations, a substantial proportion of the product was accumulated in the cyclone, especially at the lowest drying temperature.

### 3.2. Morphology Assessment

**Figure 1:**
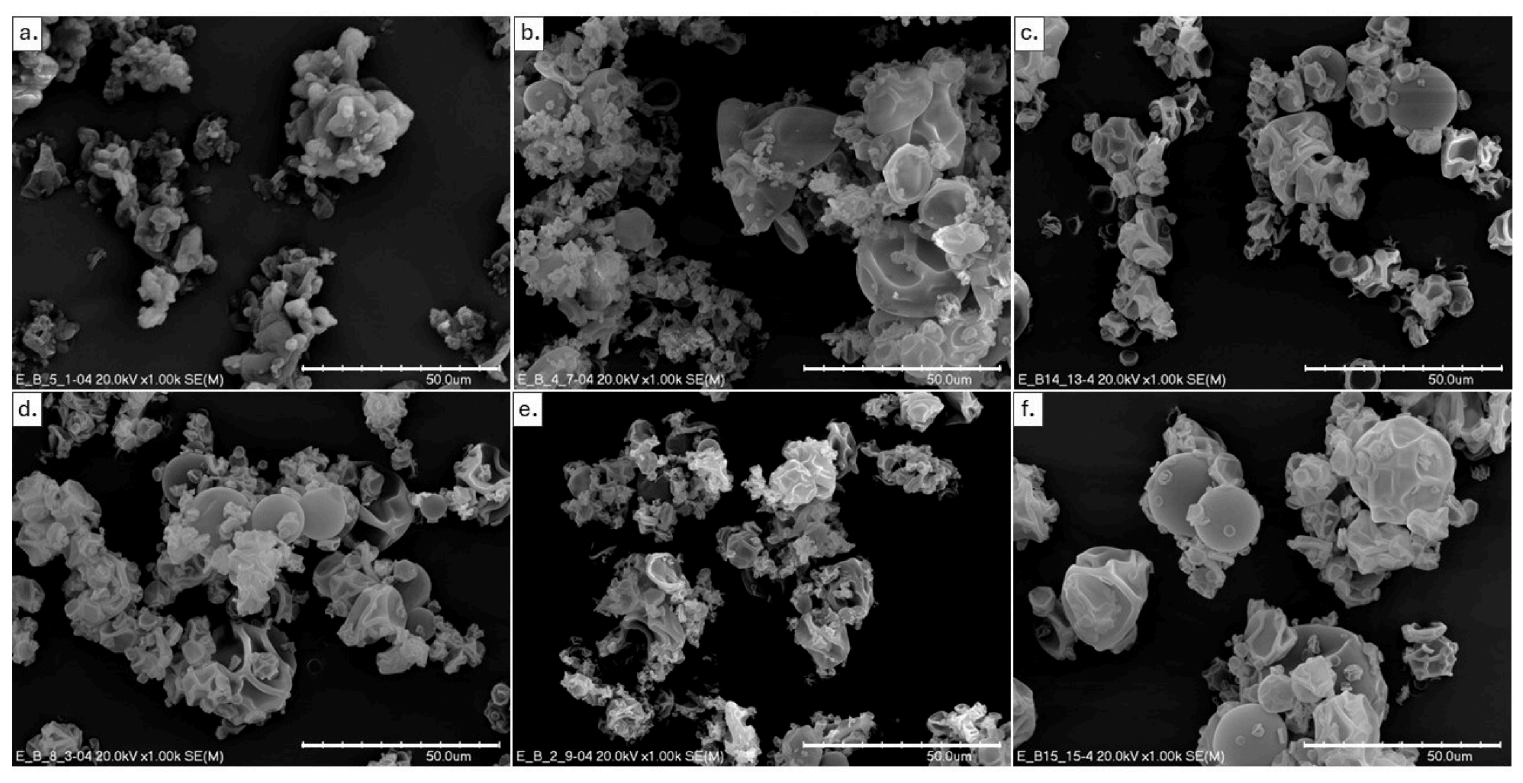
SEM micrographs of spray-dried microparticles: (**a**) BF_EC_9:1_40 °C. (**b**) BF_EC_1:1_40 °C. (**c**) BF_EC_1:9_40 °C. (**d**) BF_EC_9:1_60 °C. (**e**) BF_EC_1:1_60 °C. (**f**) BF_EC_1:9_60 °C (white scale bar indicates 50 µm).

Figure 1 presents SEM micrographs of selected microparticles dried at 40 °C (Figure 1a–c) and 60 °C (Figure 1d–f). The micrographs indicate that the majority of the obtained microparticles exhibited irregular morphologies, with collapsed and wrinkled shells. The particles were characterized by a diverse size distribution and a pronounced tendency to form agglomerates of small particles. These observations were consistent with the macroscopically observed poor flow properties of the powders produced by spray drying.

In the case of microparticles containing 90% BF, dried at 40 °C (Figure 1a) and 50 °C, an irregular particle morphology was observed. The microparticles exhibited a concave shape and a relatively smooth surface. This may suggest the formation of an outer shell during solvent evaporation, followed by its contraction or collapse as the internal droplet volume decreased. However, as TGA analysis did not indicate measurable mass loss attributable to residual volatile components in the low-temperature range, these morphological differences cannot be considered evidence of incomplete drying (Figure S1). Figure 1b,e depict microparticles containing BF and EC in a 1:1 ratio. A distinctive feature of this formulation was the presence of a substantial fraction of agglomerated fine particles, irrespective of the spray-drying temperature. In contrast, the microparticles containing 10% BF (Figure 1c,f) exhibited a slightly narrower particle size distribution; however, they continued to form irregular agglomerates.

### 3.3. PSD Analysis

**Figure 2:**
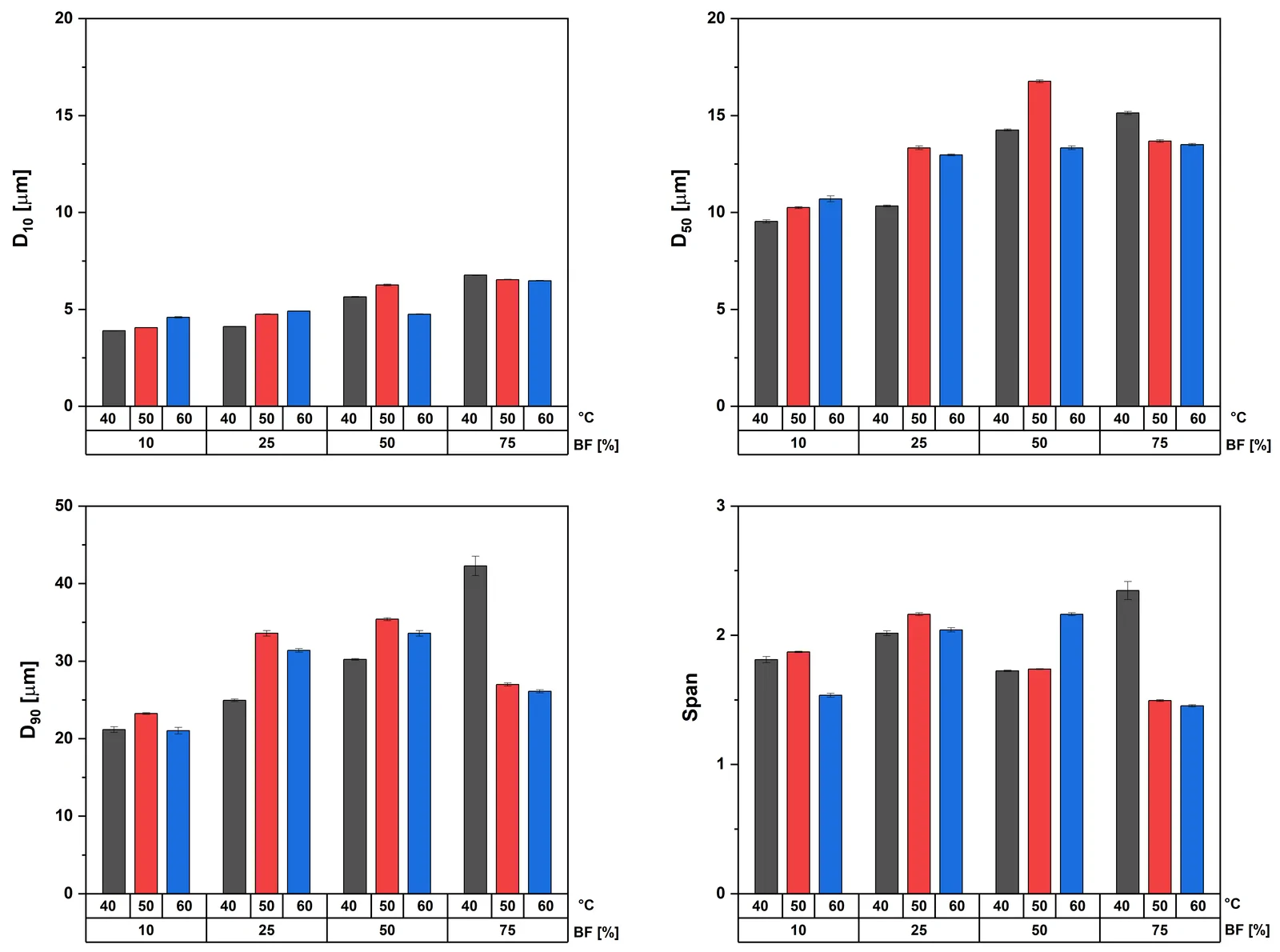
Particle size distribution parameters of prepared microparticles.

Particle size analysis revealed significant differences among the prepared microparticle formulations. The values of D_10_ ranged from 3.89 µm to 6.77 µm, D_50_ from 9.54 µm to 16.77 µm, and D_90_ from 21.02 to 42.27 µm (Figure 2). The particle size distributions were relatively narrow to moderately broad, with span values between 1.45 and 2.35, depending on the formulation.

Figure 2 indicates an increasing trend in the microparticle size with increasing BF content. In contrast, although the differences in particle size for the same formulation spray-dried at different temperatures were statistically significant (*p* < 0.05), there was no visible trend of this parameter’s influence among all compared formulations (Table S2). Significant differences in span values were found among the tested formulations, but no clear trend could be established with respect to either of the investigated process parameters.

**Figure 3:**
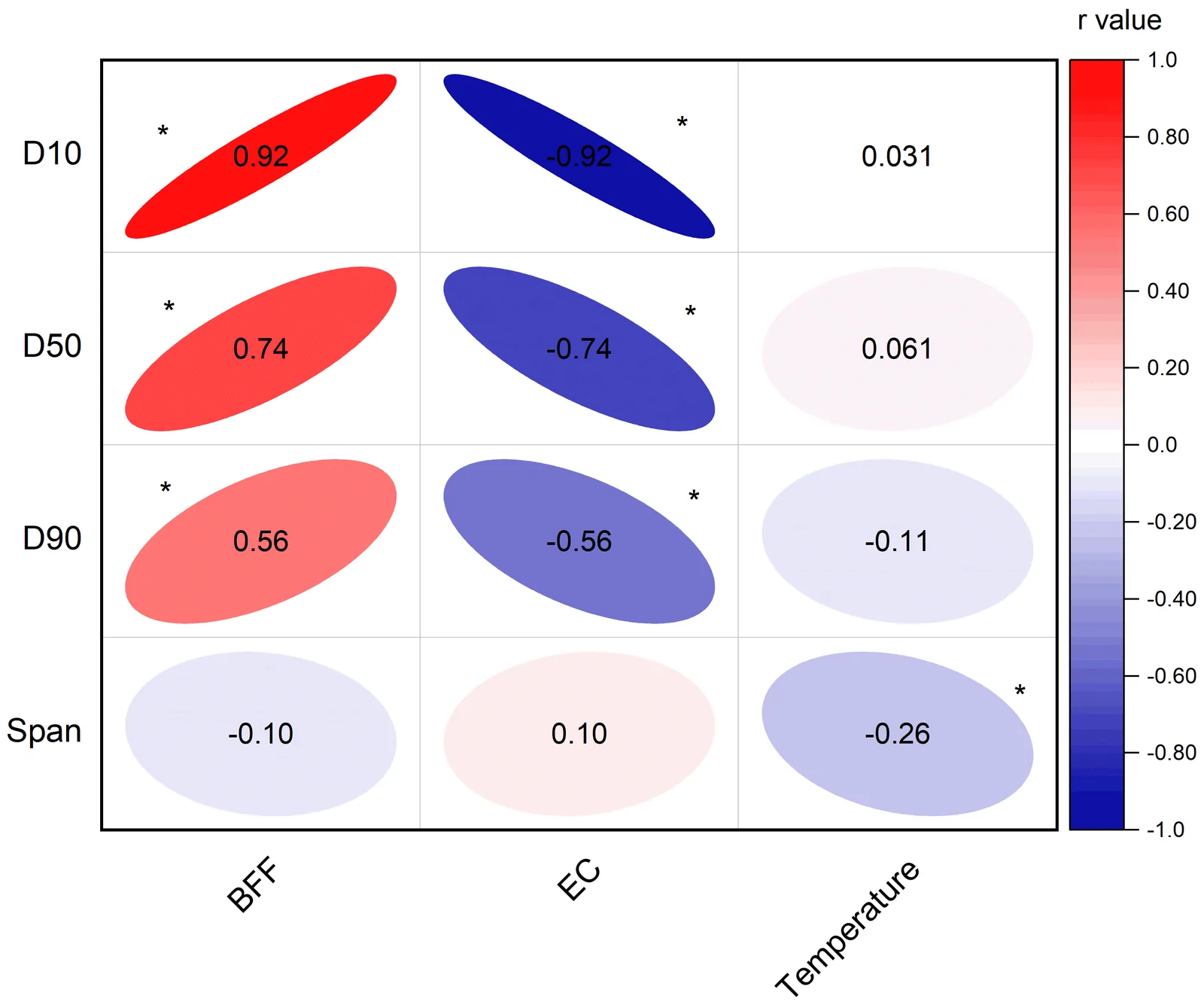
Results of Pearson correlation analysis between formulation and process parameters (BF content, EC content and temperature) and particle size distribution parameters (D_10_, D_50_, D_90_ and span). Values shown in the ellipses represent Pearson correlation coefficients (r), while asterisks (*) indicate statistical significance (*p* < 0.05).

Statistical analysis of the correlation between spray-drying process parameters and microparticles’ size descriptors indicated a strong and significant effect of formulation composition (Figure 3). A strong positive correlation was observed between BF content and D_10_ (r = 0.92), suggesting an approximately linear relationship. Slightly lower correlation coefficients were obtained for D_50_ and D_90_ (r = 0.74 and 0.56, respectively), although both remained statistically significant (*p* < 0.05). No significant correlation was observed between the span values and any of the investigated process parameters. Although the particle-size parameters varied significantly with spray-drying temperature, no consistent relationship was identified.

**Figure 4:**
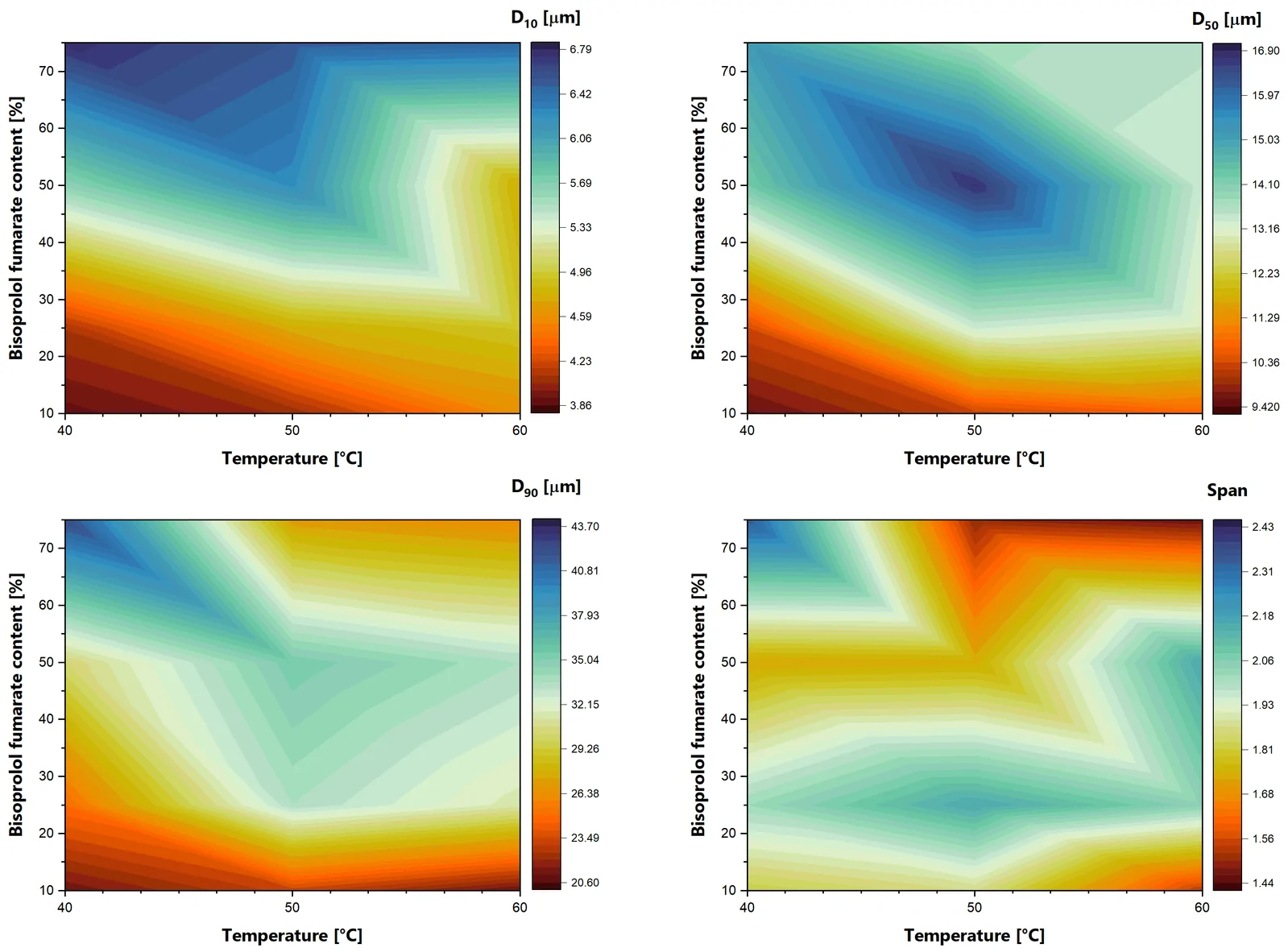
Explanatory graphical plots illustrating the effects of spray-drying temperature and BF content on particle size distribution parameters.

Explanatory graphical plots indicate a clear relationship between BF content and D_10_ values (Figure 4). An increase in BF content in the microparticle composition was associated with an increase in particle size. A similar trend was observed for D_50_ and D_90_, but the effect was less pronounced. Across the investigated formulations, the composition (polymer-to-API ratio) was the main factor governing particle size, whereas spray-drying temperature did not show any statistically significant effect on any of the particle size distribution parameters. The absence of a temperature effect also suggests a lack of significant interaction between the investigated factors.

It should be noted that the observed relationships between BF content and microparticle properties are inversely related to EC content, as an increase in BF content corresponded to a decrease in EC content in the formulations.

### 3.4. DSC and XRPD Analysis

**Figure 5:**
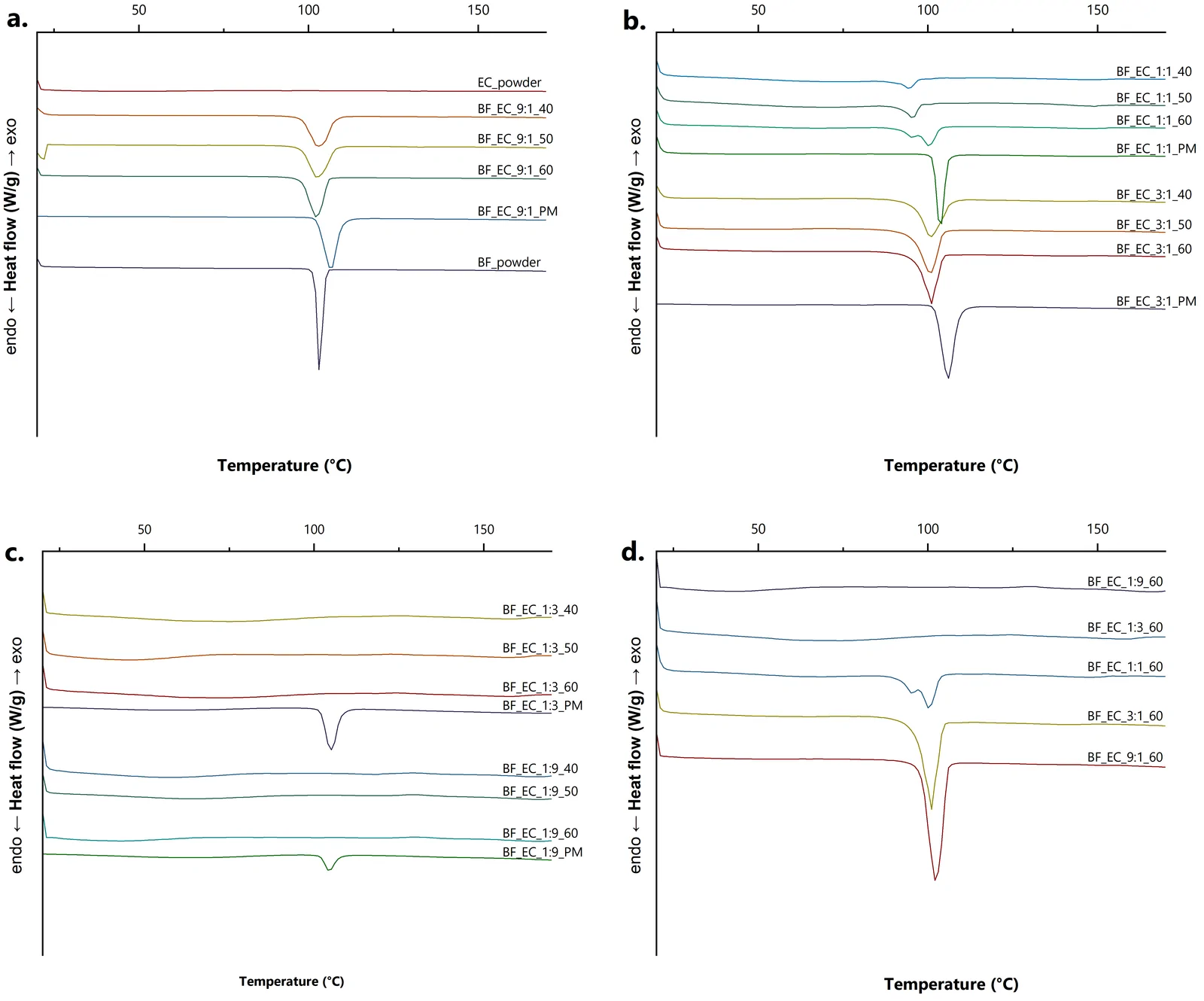
DSC profiles of (**a**) bisoprolol fumarate (BF_powder), ethylcellulose (EC_powder), and microparticles of BF_EC_9:1 formulation and corresponding physical mixture. (**b**) microparticles of BF_EC_3:1 and BF_EC_1:1 formulations and corresponding physical mixtures. (**c**) microparticles of BF_EC_1:3 and BF_EC_1:9 formulations and corresponding physical mixtures. (**d**) microparticles of all BF-to-EC ratios spray-dried at 60 °C.

**Figure 6:**
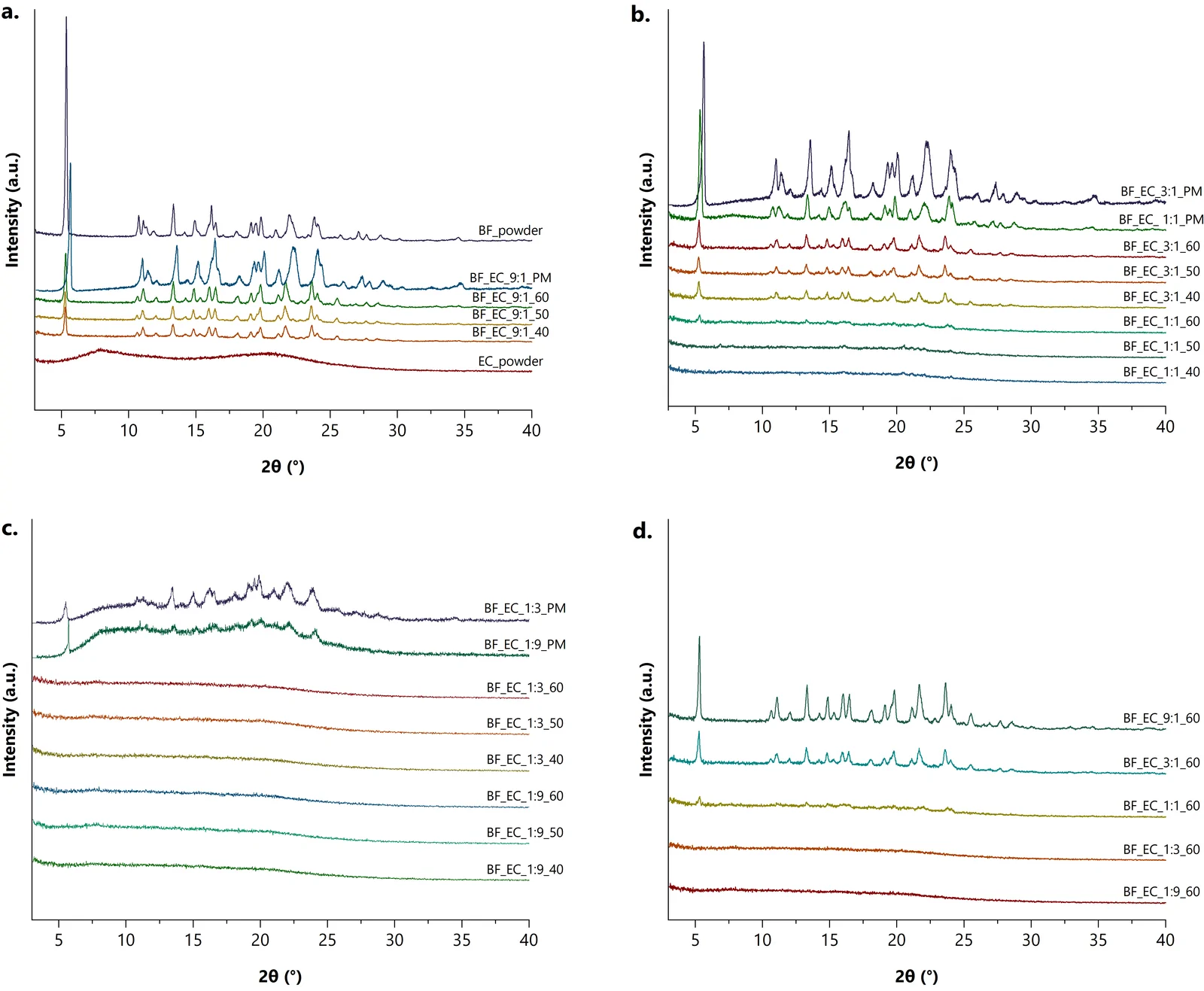
XRPD diffraction patterns of (**a**) bisoprolol fumarate (BF_powder), ethylcellulose (EC_powder), microparticles of BF_EC_9:1 formulation and corresponding physical mixture. (**b**) microparticles of BF_EC_3:1 and BF_EC_1:1 formulations and corresponding physical mixtures. (**c**) microparticles of BF_EC_1:3 and BF_EC_1:9 formulations and corresponding physical mixtures. (**d**) microparticles of all BF-to-EC ratios spray-dried at 60 °C.

Figure 5 and Figure 6 present the DSC and XRPD results obtained for the prepared microparticles, BF and EC. To date, three polymorphic forms of BF have been reported: a hydrated form, polymorph I, and polymorph II. The DSC curve of BF exhibited a sharp endothermic peak at 103.1 °C, attributed to its melting point. The DSC thermograms, supported by XRPD analysis, indicated that the BF sample analyzed in this study corresponded to polymorph I [53].

According to the literature, EC is considered a semi-crystalline polymer with a melting temperature in the range of 165–180 °C [54,55]. In this study, the DSC thermogram of EC displayed a melting event at 180.8 °C. However, the XRPD diffractogram showed two broad halos centered at approximately 8° (2θ) and 20° (2θ), characteristic of predominantly amorphous materials, without distinct diffraction peaks. This apparent discrepancy may be related to the limited XRPD scanning range (40° in 2θ). As reported by Mahnaj et al. [54], the most intense diffraction peaks for EC occur at 38.4° (2θ) and 44.5° (2θ); therefore, at least one of these characteristic reflections lies outside the upper limit of the measurement range used in this study [54].

In the case of the BF_EC_1:9 and BF_EC_1:3 formulations, complete amorphization of the API was achieved. In the DSC thermograms, no endothermic or exothermic events indicative of a crystalline phase were detected; instead, broad, low-intensity endothermic peaks below 100 °C, attributable to water or solvent evaporation, were observed. However, in the case of the BF_EC_1:9 formulation, regardless of the drying temperature, the TG analysis did not reveal any measurable mass loss in the low-temperature region, indicating that spray drying enabled the removal of nearly all residual ethanol and that the resulting powders did not contain appreciable amounts of adsorbed water (Figure S1). Likewise, in the X-ray diffraction patterns, no Bragg peaks characteristic of BF were present. Only a low-intensity amorphous halo, typical of EC, was detected, further confirming the absence of long-range crystalline order.

For the BF_EC_1:1 formulation, both DSC and XRPD analyses revealed features indicative of a semicrystalline structure of the investigated samples. The thermograms of microparticles dried at 40 °C and 50 °C exhibit characteristic endothermic peaks corresponding to BF melting (observed at 94.4 °C and 95.4 °C, respectively). These thermal events can be ascribed to BF polymorphic form II; however, they are superimposed on a broad endothermic event associated with water or solvent evaporation. These observations are confirmed by XRPD patterns, which reveal weak diffraction signals attributable to BF crystals. More intense Bragg peaks were detected in the diffractogram of microparticles dried at 60 °C, which can be ascribed to the presence of BF polymorph I. The DSC thermograms for this formulation exhibited a discontinuity in the endothermic peak associated with crystalline BF, resolving into two distinct peaks at 95.4 °C and 100.2 °C. These findings suggest the coexistence of at least two crystalline polymorphs in the sample, which could be polymorphs I and II.

For formulations BF_EC_3:1 and BF_EC_9:1, the presence of BF crystals was also confirmed. The signal intensity in the samples increased with the BF content in the microparticles. However, the XRPD signals were less intense than the diffraction peaks of the pure substance, indicating partial amorphization of the analyzed samples. The predominant polymorphic form of BF observed in the diffractograms was Form I. It was also noted that the peaks in the DSC thermograms and the XRPD patterns were most intense for microparticles dried at 60 °C. In contrast, the signals from microparticles dried at 40 °C were more diffuse and less defined, suggesting a lower structural homogeneity of the crystalline phase.

### 3.5. Drug-Loading and Dissolution Studies: Microparticles

**Figure 7:**
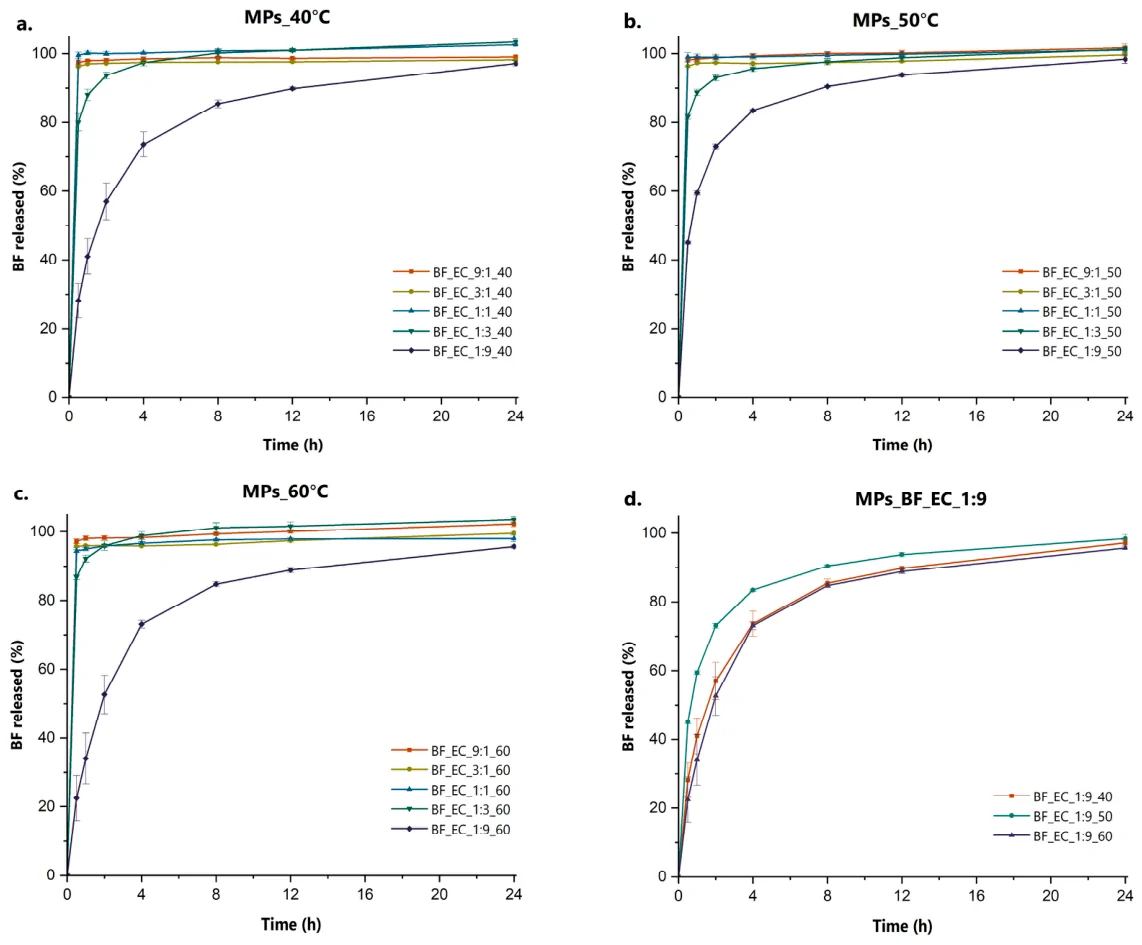
Dissolution profiles of microparticles spray-dried at (**a**) 40 °C, (**b**) 50 °C, and (**c**) 60 °C. (**d**) Dissolution profiles of BF_EC_1:9 microparticles spray-dried at 40 °C, 50 °C and 60 °C.

The drug-loading efficiency of the formulated microparticles ranged from 94.03% to 110.46% relative to the theoretical value (Table S1). Actual drug loading in the prepared microparticles ranged from 9.40% to 88.17% of the powder mass. All microparticles containing more than 10% of BF showed immediate-release characteristics (Figure 7), irrespective of the spray-drying temperature. The AUC values were comparable, ranging from 46.25 to 48.04 (Table 3 and Table S7). The amount of BF released from these formulations after 30 min was at least 80%. For microparticles with BF to EC ratios of 9:1, 3:1, and 1:1, the amount of BF released after 30 min ranged from 94.4% to 99.5%. The mean dissolution time for these formulations ranged from 0.37 h to 0.93 h. A slightly slower release profile was observed for microparticles BF_EC_1:3. Although the amount of BF released after 30 min still exceeded 80%, complete drug release was achieved only after up to 8 h. The MDT values for this formulation ranged from 0.91 h to 1.11 h (Table 4). Nonetheless, the polymer content was insufficient to provide a pronounced and sustained retardation of BF release, despite complete amorphization confirmed by DSC and XRPD analyses.

The formulation BF_EC_1:9, containing the highest proportion of EC, exhibited the most pronounced retardation of BF release compared with other formulations with immediate-release properties. However, this prolonged-release effect was relatively moderate, with a high initial release rate (Figure 7d). The amount of BF released after 2 h ranged from 52.6% to 73%, increasing to 84.8–90% after 8 h and reaching over 96% after 24 h. MDT value for this formulation ranged from 2.35 to 3.53 h (Table 4), and the AUC value ranged from 39.18 to 42.59 (Table 3). Among the samples tested, the microparticles dried at 50 °C were characterized by the fastest release of BF. Consequently, the AUC and MDT values calculated for them were significantly different from those for microparticles spray-dried at different temperatures (*p* < 0.05; Tables S7 and S9). The AUC value for BF_EC_1:9_50 was the highest among the other microparticles in this formulation (42.59), while the MDT was the lowest (2.35 h). This finding is consistent with the rapid release of BF observed during the initial stages of the dissolution study. Despite this, these microparticles still exhibited the slowest BF release among all formulations dried at 50 °C. To confirm that the prolonged-release behavior resulted from the microparticle structure rather than from the presence of EC alone, dissolution studies were also performed for the corresponding physical mixtures. All evaluated PMs exhibited immediate release of BF, with over 99% of BF released after 30 min in this study. In the case of the BF_EC_1:9 physical mixture, this observation confirms that the prolonged-release behavior was associated with the incorporation of BF within the EC matrix during microparticle formation.

**Figure 8:**
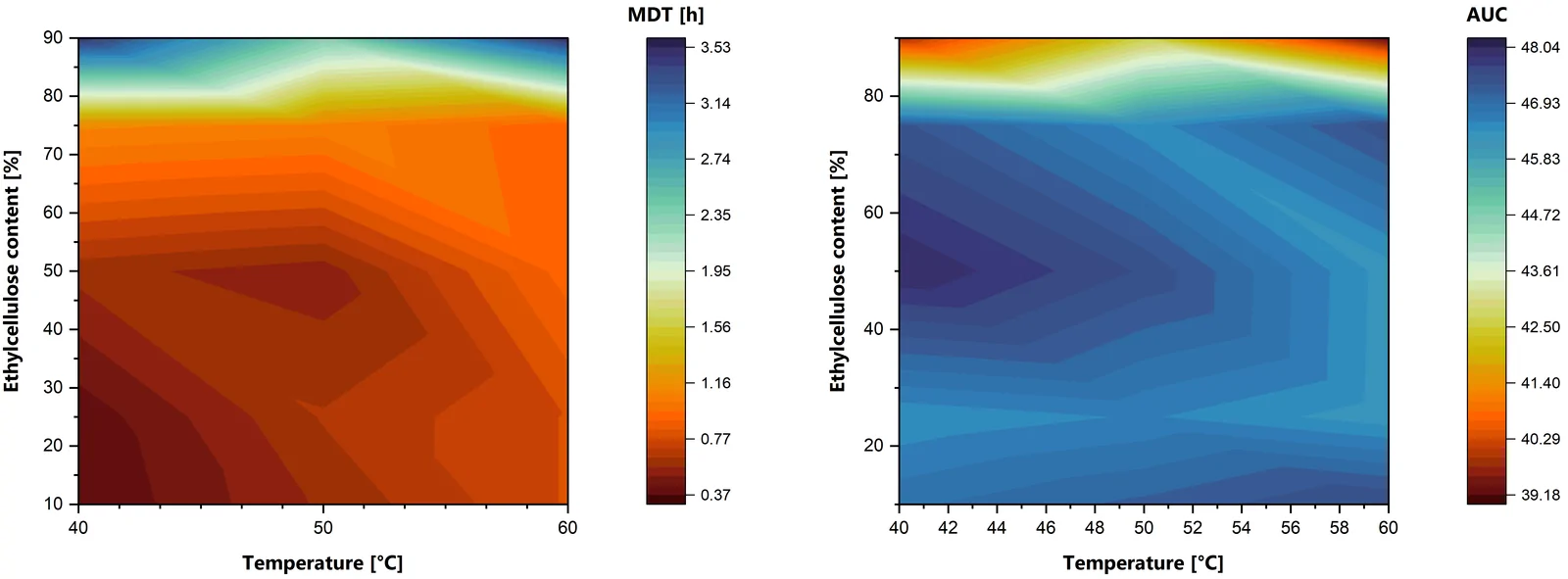
Explanatory graphical plots illustrating the effects of spray-drying temperature and EC content on the values of mean dissolution time (MDT) and area under the dissolution curve (AUC).

Correlation analysis demonstrated a significant positive correlation between the content of the EC in the microparticles and the value of MDT (r = 0.75; *p* < 0.05). An opposite and weaker correlation was found for the AUC values (r = −0.62; *p* < 0.05). In contrast, the spray-drying temperature did not significantly affect these parameters (Figure 8). Only a weak effect of temperature was observed, with a slight increase in MDT for formulations containing lower BF-to-EC ratios (i.e., a higher EC content).

### 3.6. Compression of Microparticles to Obtain Minitablets

Three types of microparticles, differing in release profiles and BF content, namely, BF_EC_9:1_60, BF_EC_1:1_60, and BF_EC_1:9_60, were subsequently compressed into minitablets. As shown in Table 2, all formulations contained constant quantities of superdisintegrants (Primojel and Primellose), fumed silica (Cab-O-Sil), and lubricant (Lubripharm). The compositions differed only in the type and proportion of microparticles to maintain a consistent BF content of 1 mg in a single minitablet. The total tablet mass was adjusted with microcrystalline cellulose (MCC) to 16 mg. Consequently, in formulation MT_BF_EC_9:1, the contents of microparticles and MCC were 6.9% and 79.9%, respectively, whereas in formulation MT_BF_EC_1:9, these values were 62.5% and 24.4%. Each formulation was compressed with four different compression forces (150, 250, 350, and 400 N), resulting in a total of 12 minitablet series.

Table 5 presents the tensile strength of the minitablets. Formulation MT_BF_EC_1:9 exhibited the highest value of this parameter (1.04–2.62 MPa). Despite the evaluation of only four different compression pressures, tensile strength showed a strong linear relationship with compression pressure (R^2^ = 0.9855). For the remaining two formulations, significantly lower tensile strength values (0.48–1.62 MPa) were obtained. In the case of formulation MT_BF_EC_1:1, containing 12.5% microparticles in the tablet blend, the relationship between tensile strength and compression pressure was also well described by a linear model (R^2^ = 0.9829). Overall, an increase in compression force (pressure) resulted in a corresponding increase in minitablet tensile strength in all formulations, although the limited number of pressure levels should be considered when interpreting the tabletability profiles.

The minitablets also differed in thickness. At a compression force of 150 N, minitablets containing prolonged-release microparticles (MT_BF_EC_1:9) had the greatest thickness (2.70 ± 0.08 mm), whereas the thickness of MT_BF_EC_9:1 and MT_BF_EC_1:1 was comparable, measuring 2.45 ± 0.15 mm and 2.50 ± 0.02 mm, respectively. Increasing the compression force resulted in a decrease in the minitablet thickness, reaching 2.34 ± 0.09 mm for MT_BF_EC_1:9 and 1.99 ± 0.02 mm for the remaining formulations.

### 3.7. Minitablet Morphology

**Figure 9:**
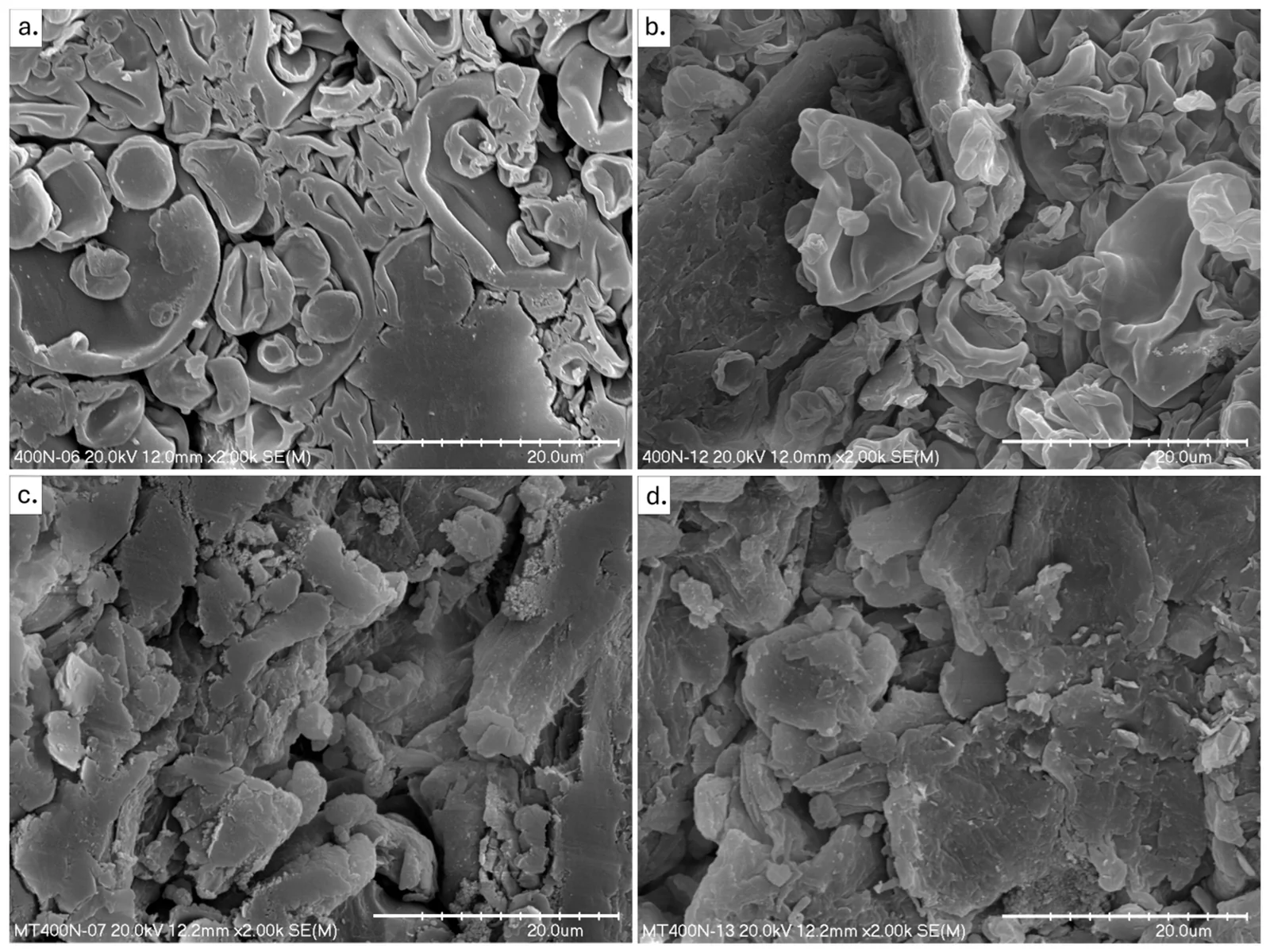
SEM micrographs of minitablets’ lateral external surface and internal fracture surface: (**a**) MT_BF_EC_1:9_400 (external surface), (**b**) MT_BF_EC_1:9_400 (fracture surface), (**c**) MT_BF_EC_9:1_400 (external surface), and (**d**) MT_BF_EC_9:1_400 (fracture surface) (white scale bar indicates 20 µm).

Figure 9a,b and Figures S2–S5 (supplementary materials) present micrographs of the lateral external surface and the internal fracture surface of formulation MT_BF_EC_1:9_400. The internal surface was exposed by manually breaking the minitablet in two parts. On the tablet external surface, flattened microparticles enclosed in the minitablet matrix are visible. In contrast, the fracture surface view (Figure 9b) reveals that the microparticles retain their original morphology and constitute the predominant fraction of the tablet mass. No structural damage to the microparticles was observed at any of the applied compaction forces, suggesting a high mechanical robustness of the obtained microparticles. In the case of formulation MT_BF_EC_9:1_400 (Figure 9c,d), the tablet blend contained a substantially lower proportion of microparticles (7%), so it was difficult to distinguish them in the tablet mass structure composed mainly of microcrystalline cellulose (MCC).

### 3.8. Minitablet Disintegration Time

The developed minitablets were designed to provide rapid disintegration with prolonged drug release. Upon disintegration, the minitablets are expected to disperse into a suspension of BF-loaded microparticles, which are intended to preserve their prolonged-release characteristics during swallowing.

The disintegration times of all minitablets obtained were below 30 s (Table 6). These results comply with the US Food and Drug Administration (FDA) recommendations for ODTs’ disintegration time, which should be less than 30 s [56]. Within each applied compression force, the formulation MT_BF_EC_9:1 exhibited the shortest disintegration time (3.4–10.5 s). MT_BF_EC_1:9 was characterized by the longest disintegration time in the range of 13.9–28.9 s. Within all minitablet formulations, the disintegration times increased with increasing compression pressure. For formulation MT_BF_EC_1:1, this increase was well described by a linear model within the tested compression force range (R^2^ = 0.9876). This empirical relationship may reflect tablet mass densification and slower penetration of water into the matrix of minitablets compressed with higher forces. However, because only four different compression forces were examined, this cannot be interpreted as a general mechanistic relationship beyond these values.

### 3.9. Dissolution Studies: Minitablets

**Figure 10:**
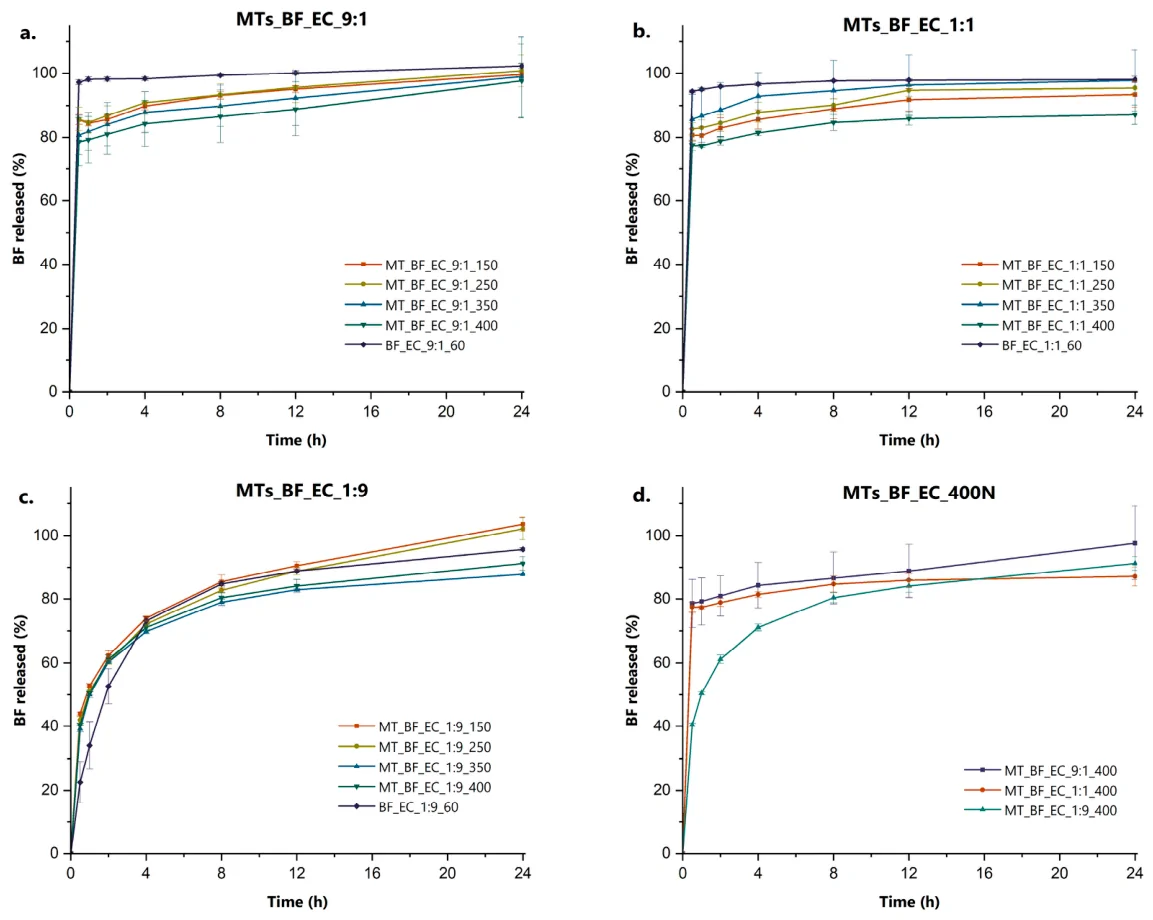
Dissolution profiles of minitablets compressed with different compression forces: (**a**) MT_BF_EC_9:1. (**b**) MT_BF_EC_1:1. (**c**) MT_BF_EC_1:9. (**d**) Dissolution profiles of minitablets compressed with 400 N.

The obtained minitablets maintained the overall drug release characteristics of the microparticles (Figure 10). The minitablets with microparticles containing the highest amount of EC were characterized by prolonged release of BF, whereas the minitablets obtained from BF_EC_9:1_60 and BF_EC_1:1_60 maintained the immediate release of BF. Despite the use of different compression forces, the minitablets obtained from the aforementioned microparticles did not differ significantly in terms of their release profiles and amount of BF released (Figure 10a–c). These observations are confirmed by the calculated MDT values, where only the MT_BF_EC_9:1_400 differed significantly from the reference microparticles (2.30 h vs. 0.77 h) (Table 7 and Table S10).

In the case of formulation MT_BF_EC_1:9 (Figure 10c), a faster drug release from the minitablets compared with the corresponding microparticles was observed during the initial phase of the study (39.1–44.0% vs. 22.5% within the first 30 min and 50.0–52.8% vs. 34.0% after 1 h, respectively). After 2 h, these differences decreased and were no longer statistically significant. The calculated similarity factor f_2_ values ranged from 45.04 to 49.23, which were below the conventional similarity criterion of 50, indicating that the dissolution profiles of the MT_BF_EC_1:9 minitablets differed from those of the corresponding bulk microparticles (Table 8). These findings suggest that compression modified the dissolution profile of the microparticles, particularly during the initial phase of the study; nevertheless, the minitablets maintained the prolonged-release characteristics of BF, releasing not more than 85% of BF for the first 8 h. The observed changes in the dissolution profile between bulk microparticles and minitablets may be related to their mixing with excipients of better wettability, rather than to the compression itself.

The minitablets compressed at 150 N and 250 N were characterized by complete release of BF, whereas the amounts of API released from the MT_BF_EC_1:9 at 350 N and 400 N were 89% and 91%, respectively. Consequently, the MDT and AUC values (Table 7 and Table 9) calculated for these formulations differed significantly from those for minitablets compressed at lower compression forces (*p* < 0.05; Table S10). Meanwhile, analysis of the f_2_ values for minitablets MT_BF_EC_1:9 compressed with different forces showed that the dissolution profiles of the individual minitablet series exhibited comparable prolonged-release characteristics. The values of this parameter were in the range of 67.6–85.6 (Table 8). This apparent discrepancy may arise because MDT represents a single summary parameter and can be sensitive to relatively small differences in release, whereas f_2_ evaluates the profiles across all selected sampling points.

For formulations MT_BF_EC_9:1 and MT_BF_EC_1:1, it was observed that, compared with the corresponding microparticles, the initial burst release of BF was significantly lower (Figure 10a,b). The amount of BF released after 30 min from microparticles was 94–97%, while for minitablets, it reached only 78–86%. These observations were confirmed by the comparison of MDT values (Table S10). This parameter was significantly higher in the case of minitablets MT_BF_EC_9:1 (1.57–2.30 h) than for the BF_EC_9:1_60 microparticles (0.77 h). The calculation of f_2_ values for these minitablets was impossible because the amount of BF released at the first time point exceeded 85%.

### 3.10. Analysis of Release Kinetics

The release profiles of prolonged-release microparticles were best described by the Peppas–Sahlin model. In contrast, BF_EC_1:9_50 and BF_EC_1:9_40 exhibited exceptionally low RMSE values (0.0002 and 0.0006, respectively), which may be due to the overparameterization of the Peppas–Sahlin model (Table 10). However, the other evaluated models had significantly higher RMSE values (>0.4) and were, therefore, considered not to adequately reflect the experimental data.

For tablets compressed at 150 N (MT_BF_EC_1:9_150), the release kinetics were most appropriately described by the Korsmeyer–Peppas model. The low release exponent (n ≈ 0.25) might indicate diffusion-limited drug release through a porous compact structure [57]. Increasing the compression force to 250 N and 350 N improved the fit to the Peppas–Sahlin model, indicating a higher degree of mechanistic complexity attributable to tablet densification and reduced penetration of the dissolution medium. For tablets compressed at 400 N, the best fit was found for the Hopfenberg model, suggesting a shift from diffusion towards erosion (Table 10).

## 4. Discussion

During the spray-drying process, it was observed that microparticle formulations containing high proportions of EC (BF_EC_1:3 and BF_EC_1:9) or the highest proportion of BF (BF_EC_9:1), respectively, exhibited reduced process efficiency across the entire evaluated temperature range compared with the other formulations.

In the case of microparticles containing a predominant amount of EC, this phenomenon can be attributed to the elevated concentration of the film-forming polymer, which promotes the formation of highly viscous droplets that tend to deposit on the surface of the drying chamber.

For BF_EC_9:1, it is likely that very fine, low-density particles of the API were generated during processing, or that the microparticles underwent fragmentation during the drying step as a consequence of their low mechanical elasticity. Consequently, a substantial fraction of the product deposited on the filters or within the cyclone, thereby reducing the overall process efficiency.

The optimal API-to-polymer ratios and drying temperatures that concurrently balanced particle solidification, surface tackiness, and aerodynamic performance during spray drying were identified for the BF_EC_3:1 and BF_EC_1:1 formulations, which were dried at 60 °C and 50 °C, respectively, and exhibited high spray-drying process yields (66.8% and 59.2%).

All of the resulting microparticles exhibited collapsed shells and a pronounced tendency to form agglomerates composed of small particles. Wasilewska et al. [58] reported a similar collapsed and irregular microparticle morphology for an organic ethylcellulose-based spray-dried formulation containing rupatadine fumarate at a drug-to-polymer ratio of 1:2. In contrast, when aqueous solutions of ethylcellulose containing plasticizers, stabilizers, and surfactants were employed, the process yielded mostly spherical particles characterized by a smooth surface, indicating that particle morphology depends not only on the polymer but also on the spray-dried liquid composition and its drying behavior [58]. The collapsed morphology observed in our study may be qualitatively explained by the Peclet number, which reflects the balance between solvent evaporation and component diffusion within a drying droplet. Because EC is expected to diffuse more slowly than BF, its component-specific Peclet number may be relatively high, promoting EC accumulation at the droplet surface and early shell formation during the spray-drying process. Continued solvent removal could subsequently cause shell contraction or buckling, resulting in the observed concave, collapsed morphology. However, because the relevant diffusion coefficients and droplet-drying kinetics were not determined, this interpretation remains only qualitative. Furthermore, the small particle size and irregular surfaces may increase cohesive interactions through the increase in interparticle contact. This seems to be consistent with poor flowability and a visible tendency to agglomerate.

The individual particle diameters were relatively small, with median values between 9.54 µm and 16.77 µm. It was found that particle size was dependent on the formulation composition, with increasing BF content leading to the formation of bigger microparticles. Although it is not a universal relationship, a similar trend was also reported in a study on spray-dried gelatin microspheres containing ganciclovir [59]. The higher drug-to-polymer ratio resulted in the preparation of particles with significantly higher particle size. This relationship may be more complex because changing the drug-to-polymer ratio may alter the viscosity of the spraying liquid, surface tension, component diffusion, particle density, and susceptibility to agglomeration. These factors can affect atomized droplet size, resulting in changes in the dimensions of the dried particles [60]. Therefore, the observed increase in particle size should be regarded as a formulation-specific effect of replacing EC with BF rather than as a general consequence of increasing drug substance content in the spray-dried liquid. Although the drying temperature affected some particle-size parameters, its effect was not consistent, whereas the influence of formulation composition showed a more clearly identifiable trend.

The particle size distribution of the resulting microparticles impacts the formulation and characteristics of the minitablets. The small particle size is likely to enhance interparticle cohesive forces, which may, in turn, diminish powder flowability and pose challenges during the preparation of tableting blends [61]. Meanwhile, smaller particles may improve compressibility and enhance resistance to breakage under compression [62]. Due to their size, they can occupy interparticulate voids between larger excipient particles and may be partially protected by the plastic deformation of the surrounding matrix [62]. In the evaluated formulations, the fine microparticles may have been accommodated between and partially surrounded by the plastically deforming MCC particles, which could have limited their exposure to direct mechanical stress. However, their exact impact on tablet porosity and water penetration cannot be predicted from particle size alone because it depends on many other factors, such as hydrophobicity, shape, the ratio of microparticles to other excipients, or deformation of MCC. Therefore, these mechanisms can only be treated as a possible explanation, not a definite conclusion.

The resulting microparticles were subjected to thermal and structural analysis. For the investigated samples, complete amorphization of the API was obtained for the BF_EC_1:9 and BF_EC_1:3 formulations, which contained the highest proportion of EC. A semicrystalline structure was confirmed in the remaining formulations; among these, the BF_EC_9:1 samples dried at 60 °C showed the highest signal intensity in both DSC and XRPD analyses, indicating the greatest proportion of crystalline BF.

The observed differences in the physical form of BF may result from the interaction of several factors related to the formulation and the process, including process temperature, the active ingredient-to-polymer ratio, and the presence of residual volatile components. A higher EC content may provide greater stabilization of the amorphous BF by limiting molecular mobility and inhibiting the molecular rearrangements necessary for crystal nucleation and growth. In contrast, in formulations with a lower EC content, particularly in BF_EC_1:1, the presence of residual volatile components may have reduced the physical stability of the amorphous matrix by acting as plasticizers. Furthermore, prolonged exposure to elevated drying temperatures may have increased the likelihood of molecular rearrangement and subsequent crystallization.

The experimentally determined drug content of the spray-dried microparticles ranged from 94% to 110% of the theoretical value; similarly, the drug-loading efficiency was within a comparable range. The drug-loading efficiency values exceeding 100% indicate that the collected microparticles contained a higher BF:EC ratio than the theoretical solution used for the spray-drying process. They do not indicate recovery of more BF than was initially introduced, since the spray-drying process yield was in the range from 22.9% to 66.8% and there was always a substantial amount of powder deposited on the walls of the drying chamber. The apparent increase in the BF content may reflect differential deposition or collection of BF-rich material during feed handling and spray drying. During processing, partial evaporation of ethanol might occur, which could lead to a deposition of EC on the walls of the beaker with a spray-drying solution. Because BF has higher solubility than EC, solvent evaporation could preferentially precipitate EC, leading to an increase in the BF-to-EC ratio in the remaining feed solution. Because the material deposited on the walls of the feed beaker and drying chamber was not analyzed, a complete component-specific mass balance could not be established. The actual drug recovery from the process ranged from 22.53% to 68.08%, which showed substantial loss of the BF during the spray drying.

No consistent correlation was identified between drug-loading efficiency and either formulation composition or drying temperature, indicating that the observed variability is more plausibly attributable to analytical uncertainty than to formulation-dependent drug degradation or loss. A prolonged release of the API was observed for formulation BF_EC_1:9, which was characterized by the highest polymer content. For the remaining formulations, a rapid release of BF was observed within the first 30 min of this study, as evidenced by short MDTs of less than 1 h.

During compression, microparticle composition mainly affected minitablet tensile strength and disintegration time, especially in prolonged-release formulations with high EC content. The prolonged disintegration of MT_BF_EC_1:9 (29 s vs. 10.5 s for MT_BF_EC_9:1 at 400 N) was primarily due to its higher microparticle and, thus, EC content. The overall ratio of EC to MCC in minitablets was substantially different amongst formulations. In the case of MT_BF_EC_1:9, it was over 2:1, and in the case of MT_BF_EC_1:1, it was 1:12, while in MT_BF_EC_9:1, it was only 1:116. The increased proportion of hydrophobic EC likely reduced surface wettability and hindered water penetration, thereby delaying the disintegration process.

MT_BF_EC_1:9 also exhibited the highest tensile strength at the same compression pressure values, indicating different compaction behavior. The relatively small EC-rich microparticles may have improved packing and interparticulate contact, while plastic deformation of EC during compression [63] may have promoted bonding with the MCC matrix. The resulting mechanically stronger minitablets could prolong disintegration. However, it should be noted that due to the significant differences in the overall composition of the three prepared minitablet formulations, particularly in terms of the EC:MCC ratio, the specific effect of EC cannot be separated from those of microparticle content and overall blend composition. Moreover, only four compression pressures were investigated; the solid fraction was not determined; and the compressibility characteristic was not deeply studied. Further research using systematically varied EC:MCC ratios and comprehensive compaction analysis would, therefore, be required to distinguish their effects on tabletability, compressibility, and compactibility. For the other formulations, the increase in disintegration time with compression force (3.4 s–10.5 s for MT_BF_EC_9:1) was likely due to the high MCC content. Although MCC is porous and promotes water uptake, higher compression forces reduce tablet porosity, limiting water penetration and slowing disintegration [64].

During the dissolution studies of the formulations MT_BF_EC_9:1 and MT_BF_EC_1:1, it was observed that the obtained MODTs preserved the immediate-release dissolution profile of BF. However, the initial burst release of BF was substantially lower compared with the corresponding microparticles, and the amount of BF released from the minitablets after 30 min was below 87%. This effect may be attributed to incomplete and delayed exposure of microparticles to the dissolution medium after tablet disintegration. In particular, the relatively small size of the microparticles (D_50_ ≈ 15 µm; D_10_ ≈ 5 µm) compared with the larger MCC particles may promote their adhesion to the MCC surface. Because MCC is extensively plastically deformed during compression, a part of the microparticles may become entrapped within the compacted MCC matrix or incorporated into larger consolidated agglomerates. Although the minitablets disintegrated in no longer than 30 s, rapid disintegration did not necessarily lead to complete de-aggregation and uniform dispersion of the individual microparticles in the dissolution medium. Separation of such agglomerates and complete wetting of microparticles may take much longer, thus limiting their immediate accessibility to the dissolution medium. Additionally, compression-induced densification of the tablet matrix and the presence of excipients may hinder liquid penetration and reduce wettability compared with the relatively hydrophilic microparticles themselves, thereby delaying BF release from a part of the microparticles. Although MCC promotes tablet disintegration through capillary wicking, it was reported that it can also retard dissolution, especially when compressed with higher pressures [65].

In contrast, MT_BF_EC_1:9 was characterized by faster BF release during the 2 h of the dissolution study compared with corresponding microparticles. Despite this difference, dissolution profiles for these minitablets still showed prolonged-release characteristics, releasing 85% of BF for around 8 h, and achieving complete release no earlier than 24 h. The increased initial release rate may be attributed to the presence of excipients facilitating water penetration through the capillary network into the tablet matrix of the tablet, thereby enhancing the wettability of the microparticles and promoting BF release. If the increase in the initial BF release from MODTs was caused by microparticle damage during compression, the drug release profile would be expected to depend on the compression force. Specifically, a higher compression force should result in greater microparticle breakage and, as a consequence, a more pronounced increase in drug release. However, such a relationship was not observed. The dissolution profiles of the individual minitablet series were very similar to each other and were essentially independent of the compression force applied. The calculated f_2_ values for all MODTs containing prolonged-release microparticles BF_EC_1:9 were in the range of 67.6–85.6. The comparable MDT values also suggest that drug release was governed primarily by the intrinsic properties of the microparticles, while the tablet matrix mainly affected the initial phase of release by modulating microparticle accessibility and wettability (Table S10). Moreover, no evidence of microparticle damage was found in the SEM images of the minitablet outer surface or internal fracture surface. Based only on SEM images, we cannot completely exclude minor microscopic structural changes that are below the resolution of the applied imaging method, but this is consistent with the known characteristics of ethylcellulose, which tends to undergo deformation rather than brittle fracture under compression [63,66,67,68]. Consequently, the developed microparticles BF_EC_1:9 are expected to retain their structural integrity during tableting rather than undergo fragmentation under compression and are suitable for incorporation into tablet formulations.

The analysis of release kinetics indicated that the dissolution profiles of the prolonged-release microparticles were most accurately characterized by the Peppas–Sahlin kinetic model. This semi-empirical model characterizes complex drug-release kinetics governed by the concurrent contribution of Fickian diffusion and anomalous (non-Fickian) transport mechanisms [69,70]. The equation calculated for microparticles MT_BF_EC_1:9 indicated that the release process was predominantly controlled by Fickian diffusion, with a comparatively minor contribution from polymer relaxation or swelling. This finding is consistent with the presence of ethylcellulose, a water-insoluble, hydrophobic matrix-forming polymer that modulates drug release primarily by restricting penetration of the dissolution medium and increasing the diffusional path for the dissolved drug [71]. The limitations of this analysis involve the possibility of overparametrization of the Peppas–Sahlin model, where three adjustable parameters are matched with three data points selected for the analysis. The mechanistic references to the dissolution profiles are, therefore, speculative and would require more work in the future. However, even temporarily disregarding the Peppas–Sahlin model, we could conclude that diffusion is strongly indicated as an important drug release mechanism, and only at higher compression forces for minitablets might erosion come into play.

The obtained results suggest that drug release from the EC-based microparticles might be governed primarily by diffusion. Following incorporation into the minitablets, the release kinetics became more complex; however, the overall dissolution profiles were not substantially affected by the applied compression force.

It should also be noted that all dissolution studies were performed using only purified water. Its selection was based on the USP monograph for bisoprolol tablets and was supported by the very high aqueous solubility of BF and the generally pH-independent behavior of EC. However, purified water is not a biorelevant dissolution medium and does not reproduce the pH, ionic strength, buffer composition, or other conditions encountered in the gastrointestinal tract. Therefore, the exclusive use of water represents a limitation of this study. Future studies in simulated gastric and intestinal media would be valuable for confirming the prolonged-release behavior under more physiologically relevant conditions.

Another point worth mentioning is that the prolonged-release formulation developed in the present study was designed as a technological proof of concept, with 1 mg of BF per minitablet unit. This strength allows the dose to be adjusted in 1 mg increments by administering an appropriate number of minitablets, providing a degree of dose flexibility. However, because pediatric starting doses may be as low as 0.05 mg/kg, a 1 mg unit may not provide sufficient dosing resolution for infants and younger children. Future studies should, therefore, investigate minitablets with lower doses, such as 0.10 or 0.25 mg BF per unit, which could enable more precise dose adjustment for the wider targeted group of patients. Thus, the present study demonstrates the technological feasibility of 1 mg prolonged-release MODTs as a proof-of-concept formulation, rather than as a complete clinical solution for the administration of BF to pediatric patients.

Additionally, it should be emphasized that the relatively low BF recovery, especially in the case of BF_EC_1:9 microparticles, represents an important limitation of the spray-drying process and should be considered when assessing its potential for scale-up and manufacturing. Further optimization of feed composition and spray-drying conditions, together with a complete component-specific mass balance, would be required to improve process efficiency and establish the manufacturing feasibility of the developed formulation. Further research should also address dose and content uniformity, low BF recovery, taste masking, the acceptability of administering multiple units, and pharmacokinetic performance in appropriate animal models and humans.

## 5. Conclusions

This study demonstrated the feasibility of producing EC microparticles containing BF, a highly water-soluble drug, by spray drying and incorporating them into 3 mm MODTs. Formulation composition had a greater and more consistent influence on microparticle properties than drying temperature in the range of 40–60 °C. The highest process yields were obtained for formulations containing BF and EC in the 3:1 ratio dried at 60 °C and in the 1:1 ratio dried at 50 °C, whereas formulations with the highest BF or EC proportions showed greater product losses. All powders consisted of fine, irregular, collapsed, and cohesive microparticles. Increasing EC content led to BF amorphization and retarded drug release, although only microparticles with a 1:9 BF-to-EC ratio showed a moderately prolonged-release profile, with approximately 85% of BF released within 8 h and complete release after approximately 24 h.

Three kinds of microparticles were successfully compressed into mechanically robust MODTs that disintegrated within 30 s. In the case of microparticles with prolonged-release properties, compression altered the initial release of BF but did not eliminate the moderately prolonged-release behavior. Dissolution profiles of minitablets compressed at different pressures remained similar, and SEM observations did not reveal evident microparticle damage, suggesting that the EC-rich microparticles retained their integrity during tableting. BF release was probably governed mainly by diffusion through the EC matrix, although this mechanistic interpretation requires further confirmation. The developed 1 mg MODTs should be regarded as a proof of concept rather than a complete pediatric dosage solution. Further studies may include optimization of spray-drying conditions, dissolution in biorelevant media, decreasing BF dose in one minitablet, evaluation of taste-masking properties and acceptability, and pharmacokinetic assessments.

## Figures and Tables

**Table 1:** The composition and process temperature of the spray-dried formulations.

Formulation Name	Amount of BF [%]	Amount of EC [%]	Temperature [°C]
BF_EC_9:1_40	90	10	40
BF_EC_9:1_50	90	10	50
BF_EC_9:1_60	90	10	60
BF_EC_3:1_40	75	25	40
BF_EC_3:1_50	75	25	50
BF_EC_3:1_60	75	25	60
BF_EC_1:1_40	50	50	40
BF_EC_1:1_50	50	50	50
BF_EC_1:1_60	50	50	60
BF_EC_1:3_40	25	75	40
BF_EC_1:3_50	25	75	50
BF_EC_1:3_60	25	75	60
BF_EC_1:9_40	10	90	40
BF_EC_1:9_50	10	90	50
BF_EC_1:9_60	10	90	60

**Table 2:** The composition of minitablets containing microparticles with BF.

Ingredient	Amount in Minitablet Mass [%]		
	MT_BF_EC_9:1	MT_BF_EC_1:1	MT_BF_EC_1:9
Microparticles	6.94	12.50	62.50
(BF content)	(6.25)	(6.25)	(6.25)
(EC content)	(0.69)	(6.25)	(56.25)
Vivapur 101	79.94	74.38	24.38
Primellose	4.69	4.69	4.69
Primojel	4.69	4.69	4.69
Cab-O-Sil	1.87	1.87	1.87
Lubripharm	1.87	1.87	1.87

**Table 3:** Area under the dissolution curve (AUC) values calculated for microparticles (T—spray-drying temperature).

Formulation Name	AUC		
	T 40 °C	T 50 °C	T 60 °C
BF_EC_9:1	46.89 (±0.17)	47.17 (±0.36)	47.52 (±0.25)
BF_EC_3:1	46.40 (±0.08)	46.59 (±0.15)	46.26 (±0.07)
BF_EC_1:1	48.04 (±0.17)	47.46 (±0.37)	46.28 (±0.20)
BF_EC_1:3	47.36 (±0.39)	46.45 (±0.43)	47.44 (±0.56)
BF_EC_1:9	39.89 (±0.75)	42.59 (±0.28)	39.18 (±0.26)

**Table 4:** Mean dissolution times (MDTs) for microparticles (T—spray-drying temperature).

Formulation Name	MDT [h]		
	T 40 °C	T 50 °C	T 60 °C
BF_EC_9:1	0.37 (±0.03)	0.58 (±0.07)	0.77 (±0.09)
BF_EC_3:1	0.39 (±0.02)	0.64 (±0.02)	0.77 (±0.11)
BF_EC_1:1	0.59 (±0.03)	0.53 (±0.04)	0.93 (±0.06)
BF_EC_1:3	1.11 (±0.03)	1.07 (±0.03)	0.91 (±0.07)
BF_EC_1:9	3.48 (±0.23)	2.35 (±0.13)	3.53 (±0.26)

**Table 5:** Tensile strengths of the minitablets (CF—compression force).

Formulation Name	Tensile Strength [MPa]			
	CF 150 N	CF 250 N	CF 350 N	CF 400 N
MT_BF_EC_9:1	0.53 (±0.06)	0.66 (±0.03)	1.13 (±0.09)	1.49 (±0.07)
MT_BF_EC_1:1	0.48 (±0.04)	0.82 (±0.13)	1.45 (±0.12)	1.62 (±0.13)
MT_BF_EC_1:9	1.04 (±0.06)	1.78 (±0.07)	2.49 (±0.04)	2.62 (±0.10)

**Table 6:** Disintegration times of the minitablets (CF—compression force).

Formulation Name	Disintegration Time [s]			
	CF 150 N	CF 250 N	CF 350 N	CF 400 N
MT_BF_EC_9:1	3.40 (±0.80)	4.51 (±0.25)	7.92 (±0.52)	10.51 (±1.16)
MT_BF_EC_1:1	5.01 (±0.99)	8.16 (±0.92)	12.76 (±1.08)	13.65 (±0.78)
MT_BF_EC_1:9	13.86 (±3.72)	21.17 (±3.34)	21.51 (±1.87)	28.89 (±3.47)

**Table 7:** Mean dissolution times (MDTs) of minitablets (CF—compression force).

Formulation Name	MDT [h]			
	CF 150 N	CF 250 N	CF 350 N	CF 400 N
MT_BF_EC_9:1	1.57 (±0.12)	1.65 (±0.11)	1.87 (±0.84)	2.30 (±0.43)
MT_BF_EC_1:1	1.16 (±0.11)	1.13 (±0.19)	0.96 (±0.06)	0.95 (±0.37)
MT_BF_EC_1:9	4.07 (±0.10)	4.22 (±0.37)	2.78 (±0.12)	3.11 (±0.08)

**Table 8:** Values of similarity factor f_2_ calculated for the minitablets MT_BF_EC_1:9 compressed with different forces and bulk microparticles BF_EC_1:9_60 (for better clarity of the table, the “BF_EC_1:9” code was removed from the tested formulation names).

Reference Formulation	Test Formulation			
	MT_150 N	MT_250 N	MT_350 N	MT_400 N
Microparticles BF_EC_1:9	45.04	47.50	49.23	48.46
MT_BF_EC_1:9_150	-	81.80	67.60	73.37
MT_BF_EC_1:9_250	81.80	-	73.45	79.98
MT_BF_EC_1:9_350	67.60	73.45	-	85.58

**Table 9:** Area under the dissolution curve (AUC) values calculated for minitablets (CF—compression force).

Formulation Name	AUC			
	CF 150 N	CF 250 N	CF 350 N	CF 400 N
MT_BF_EC_9:1	44.70 (±0.41)	45.04 (±2.01)	43.65 (±4.07)	42.32 (±4.19)
MT_BF_EC_1:1	42.65 (±1.61)	43.69 (±0.94)	40.69 (±3.69)	40.17 (±0.95)
MT_BF_EC_1:9	41.29 (±0.72)	40.41 (±0.64)	37.26 (±0.20)	38.10 (±0.82)

**Table 10:** Comparison of root-mean-square error (RMSE) values for investigated kinetic models of drug release from the microparticles and minitablets.

Formulation Name	RMSE Values			
	Hixson–Crowley	Hopfenberg	Korsmeyer–Peppas	Peppas–Sahlin
BF_EC_1:9_40	0.9872	0.5748	0.4201	0.0006
BF_EC_1:9_50	0.9870	0.6274	0.4413	0.0002
BF_EC_1:9_60	0.1897	0.9822	0.1591	0.0025
MT_BF_EC_1:9_150	0.9879	0.4831	0.1864	0.1866
MT_BF_EC_1:9_250	1.1562	0.2994	0.3591	0.0444
MT_BF_EC_1:9_350	1.1708	0.2838	0.3741	0.0018
MT_BF_EC_1:9_400	1.1265	0.2511	0.3470	0.3470

## Data Availability

The data presented in this study are contained within this article and the Supplementary Materials. Further inquiries can be directed to the corresponding author.

## Supplementary Materials

The following supporting information can be downloaded at https://www.mdpi.com/article/10.3390/pharmaceutics18091080/s1. Figure S1: Thermogravimetric profiles of (a) neat BF and EC and the BF_EC_9:1, 1:1, and 1:9 systems spray-dried at (b) 313 K, (c) 323 K, and (d) 333 K. The thermograms were used to assess the thermal behavior of the components and spray-dried microparticles, including the presence of low-temperature mass loss potentially attributable to residual solvent or moisture. Figure S2: SEM image of lateral external surface of MT_BF_EC_1:9_350 N at 2000× magnification, showing the external morphology of the minitablet after compression. Figure S3: SEM image of fracture surface of MT_BF_EC_1:9_350 N at 1000× magnification, showing the internal structure of the compressed minitablet and the distribution of microparticles within the tablet matrix. Figure S4: SEM image of fracture surface of MT_BF_EC_1:9_350 N at 2000× magnification, providing a higher-magnification view of the internal structure of the compressed minitablet. Figure S5: SEM image of fracture surface of MT_BF_EC_1:9_400 N at 1000× magnification, showing the internal structure of the compressed minitablet and the distribution of microparticles within the tablet matrix. Table S1: Properties of spray-dried microparticles: drug-loading parameters and process yield. Table S2: Statistical analysis parameters for particle size distribution: D50 values. The table presents the results of one-way ANOVA, including degrees of freedom (df), F-values, and *p*-values. Table S3. Statistical analysis parameters for area under the dissolution curve (AUC) values calculated for microparticles. The table presents the results of one-way ANOVA, including degrees of freedom (df), F-values, and *p*-values. Table S4. Statistical analysis parameters for area under the dissolution curve (AUC) values calculated for minitablets. The table presents the results of one-way ANOVA, including degrees of freedom (df), F-values, and *p*-values. Table S5. Statistical analysis parameters for mean dissolution time (MDT) values calculated for microparticles. The table presents the results of one-way ANOVA, including degrees of freedom (df), F-values, and *p*-values. Table S6. Statistical analysis parameters for mean dissolution time (MDT) values calculated for minitablets. The table presents the results of one-way ANOVA, including degrees of freedom (df), F-values, and *p*-values. Table S7. Results of Tukey’s post hoc multiple-comparison test for area under the dissolution curve (AUC) values obtained for microparticles. The table presents pairwise comparisons between the investigated formulations, including 95% confidence intervals. Statistical significance was assumed at α = 0.05. Table S8. Results of Tukey’s post hoc multiple-comparison test for area under the dissolution curve (AUC) values obtained for minitablets. The table presents pairwise comparisons between the investigated formulations, including 95% confidence intervals. Statistical significance was assumed at α = 0.05. Table S9. Results of Tukey’s post hoc multiple-comparison test for mean dissolution time (MDT) values obtained for microparticles. The table presents pairwise comparisons between the investigated formulations, including 95% confidence intervals. Statistical significance was assumed at α = 0.05. Table S10. Results of Tukey’s post hoc multiple-comparison test for mean dissolution time (MDT) values obtained for minitablets. The table presents pairwise comparisons between the investigated formulations, including 95% confidence intervals. Statistical significance was assumed at α = 0.05.

## Abbreviations

The following abbreviations are used in this manuscript:

BF	Bisoprolol Fumarate
EC	Ethylcellulose
SEM	Scanning Electron Microscopy
XRD	X-Ray Diffraction
DSC	Differential Scanning Calorimetry
MPs	Microparticles
MODTs	Orodispersible Minitablets
PLA	Poly(Lactic Acid)
PLGA	Poly(Lactic Acid–Glycolic Acid) Copolymer
API	Active Pharmaceutical Ingredient
CF	Compression Force
T	Temperature

## References

[1] Adepu S., Ramakrishna S. (2021). Controlled Drug Delivery Systems: Current Status and Future Directions. Molecules.

[2] Drapińska P., Chałupka J., Skulmowska-Polok K., Sikora A. (2025). Sustained-Release Oral Delivery of NSAIDs and Acetaminophen: Advances and Recent Formulation Strategies—A Systematic Review. Pharmaceutics.

[3] Lengyel M., Kállai-Szabó N., Antal V., Laki A.J., Antal I. (2019). Microparticles, Microspheres, and Microcapsules for Advanced Drug Delivery. Sci. Pharm..

[4] da Silva R.Y.P., de Menezes D.L.B., Oliveira V.daS., Converti A., de Lima Á.A.N. (2023). Microparticles in the Development and Improvement of Pharmaceutical Formulations: An Analysis of In Vitro and In Vivo Studies. Int. J. Mol. Sci..

[5] Vlachopoulos A., Karlioti G., Balla E., Daniilidis V., Kalamas T., Stefanidou M., Bikiaris N.D., Christodoulou E., Koumentakou I., Karavas E. (2022). Poly(Lactic Acid)-Based Microparticles for Drug Delivery Applications: An Overview of Recent Advances. Pharmaceutics.

[6] Al-Khattawi A., Bayly A., Phillips A., Wilson D. (2018). The Design and Scale-up of Spray Dried Particle Delivery Systems. Expert Opin. Drug Deliv..

[7] Baumann J.M., Adam M.S., Wood J.D. (2021). Engineering Advances in Spray Drying for Pharmaceuticals. Annu. Rev. Chem. Biomol. Eng..

[8] Miller D.A., Ellenberger D., Porfirio T., Gil M., Williams R.O., Davis D.A., Miller D.A. (2022). Spray-Drying Technology. Formulating Poorly Water Soluble Drugs.

[9] Larochelle P., Tobe S.W., Lacourcière Y. (2014). β-Blockers in Hypertension: Studies and Meta-Analyses over the Years. Can. J. Cardiol..

[10] Kiuchi S., Ikeda T. (2022). Management of Hypertension Associated with Cardiovascular Failure. J. Cardiol..

[11] Muresan L., Cismaru G., Muresan C., Rosu R., Gusetu G., Puiu M., Mada R.O., Martins R.P. (2022). Beta-Blockers for the Treatment of Arrhythmias: Bisoprolol—A Systematic Review. Ann. Pharm. Fr..

[12] Mancia G., Kreutz R., Brunström M., Burnier M., Grassi G., Januszewicz A., Muiesan M.L., Tsioufis K., Agabiti-Rosei E., Algharably E.A.E. (2023). 2023 ESH Guidelines for the management of arterial hypertension. J. Hypertens..

[13] Cammarata S.M., Capone G., Lombardi N., Pani L., Mugelli A. (2022). Systematic Data Monitoring and Analysis of Cardiovascular Off-Label Prescriptions in Pediatrics: Focus on Angiotensin-Converting Enzyme Inhibitors (ACE-I) and Beta Blockers. High Blood Press. Cardiovasc. Prev..

[14] Bueno Gomez A., Healy M., Delle Donne G., Bentley S., Sukeshi M., Daubeney P., Naqvi N., Till J., Wong L. (2023). P10 Initiation of bisoprolol in paediatric patients—Experience from a specialist paediatric cardiac centre. Arch. Dis. Child..

[15] Domingues C., Jarak I., Veiga F., Dourado M., Figueiras A. (2023). Pediatric Drug Development: Reviewing Challenges and Opportunities by Tracking Innovative Therapies. Pharmaceutics.

[16] Bakheit A.H., Ali R., Alshahrani A.D., El-Azab A.S., Brittain H.G. (2021). Bisoprolol: A Comprehensive Profile. Profiles of Drug Substances, Excipients and Related Methodology.

[17] Charoo N.A., Shamsher A.A.A., Lian L.Y., Abrahamsson B., Cristofoletti R., Groot D.W., Kopp S., Langguth P., Polli J., Shah V.P. (2014). Biowaiver Monograph for Immediate-Release Solid Oral Dosage Forms: Bisoprolol Fumarate. J. Pharm. Sci..

[18] Ilyas S., Maheen S., Andleeb M., Khan H.U., Shah S., Abbas G., Shabbir S., Sher M., Masood S.A., Shafqat S.S. (2023). Development, Optimization and in Vitro-in Vivo Evaluation of Solid Lipid Microparticles for Improved Pharmacokinetic Profile of Bisoprolol. Mater. Today Commun..

[19] Sharma R., Sharma U., Paswan S.K. (2023). Aloe Vera Nanoparticles Loaded with Antihypertensive Beta-Blockers of Different Half-Life: Entrapment Efficiency and Release Behavior. Indian J. Pharm. Sci..

[20] Elhabal S.F., Mahfouz O.M., Elrefai M.F.M., Teaima M.H., Abdalla A., El-Nabarawi M. (2025). Sustained Intraocular Pressure Reduction Using Bisoprolol-Loaded PLGA Nanoparticles: A Promising Strategy for Enhanced Ocular Delivery with Reduced GFAP Expression Indicative of Lower Glial Activation. Pharmaceutics.

[21] Gupta Y., Sharma A., Vikas, Gupta R.K. (2024). Development and Optimization of Carvedilol Microparticles. Int. J. Health Adv. Clin. Res..

[22] Stojmenovski A., Gatarić B., Vučen S., Railić M., Krstonošić V., Kukobat R., Mirjanić M., Škrbić R., Račić A. (2024). Formulation and Evaluation of Polysaccharide Microparticles for the Controlled Release of Propranolol Hydrochloride. Pharmaceutics.

[23] Panainte A.D., Popa G., Pamfil D., Butnaru E., Vasile C., Tarțău L.M., Gafițanu C. (2018). In Vitro Characterization of Polyvinyl Alcohol/Chitosan Hydrogels as Modified Release Systems for Bisoprolol. Farmacia.

[24] Panainte A.D., Gafitanu C., Stoleriu I., Tarțău L.M., Popescu M.C., Lisa G., Popa E.G. (2019). New Modified Release Tablets of Bisoprolol Fumarate for the Treatment of Hypertension: Characterization and in Vitro Evaluation. J. Drug Deliv. Sci. Technol..

[25] Grzejdziak A., Brniak W., Lengier O., Żarek J.A., Hliabovich D., Mendyk A. (2024). Application of Hydrophilic Polymers to the Preparation of Prolonged-Release Minitablets with Bromhexine Hydrochloride and Bisoprolol Fumarate. Pharmaceutics.

[26] Kadioğlu E.B., Kerimoğlu O. (2025). Preparation and Characterization of Disintegrating Film Formulations Containing Bisoprolol Fumarate for the Treatment of Hypertension. J. Res. Pharm..

[27] Srebro J., Łyszczarz E., Brniak W., Majda D., Mendyk A. (2025). Evaluation of the Effect of Formulation Composition and Physicochemical Properties of Omeprazole and Bisoprolol Hemifumarate on Electrospun Nanofibers Characteristics. Nanotechnol. Sci. Appl..

[28] Ibrahim A.M., Almutairi S.K., Gamal M.M. (2026). Optimization and Evaluation of Different Fast Acting Tablet Formulations Containing Bisoprolol Fumarate. Saudi Pharm. J..

[29] Pang Y., Jia W., Wang L., Zhang Y., Gong K., Fang L. (2024). Innovative Long-Acting Bisoprolol Patch: Synergistic Ion-Pair Skin Adsorption for Drug Delivery Control Coupled with Dynamic Modulation of Penetration Enhancers. Mol. Pharm..

[30] Teaima M.H., Mohamed M.A.A., El Rehem R.T.A., Tayel S.A., El-Nabarawi M.A., Fouad S.A. (2021). Enhanced Transdermal Delivery of Bisoprolol Hemifumarate via Combined Effect of Iontophoresis and Chemical Enhancers: Ex Vivo Permeation/in Vivo Pharmacokinetic Studies. Pharmaceutics.

[31] Gul R., Khan Y., Aman T. (2022). Formulation and Evaluation of Bisoprolol Hemifumarate Emulgel for Transdermal Drug Delivery. Dissolut. Technol..

[32] Shivakumar H.N., Reddy A., Avlani D., Alayadan P., Kumar A. (2025). Design and Evaluation of Bisoprolol Fumarate Transdermal Film: A Promising Approach for Cardiovascular Therapy. J. Sci. Soc..

[33] Nozaki A., Kobayashi T., Naruhashi K., Okugawa H., Horiuchi N., Nakanishi H., Kobayashi Y., Nakamura S. (2021). An Attempt at Administering a Transdermal Formulation of Bisoprolol in Patients with Acute Cardiac Symptoms. Am. J. Emerg. Med..

[34] El-Masry S.M., Helmy S.A., Helmy S.A.M., Mazyed E.A. (2023). Patient-Friendly Extemporaneous Formulation of Bisoprolol: Application to Stability and Bioavailability Studies. Drug Deliv. Transl. Res..

[35] Khan D., Kirby D., Bryson S., Shah M., Rahman Mohammed A. (2022). Paediatric Specific Dosage Forms: Patient and Formulation Considerations. Int. J. Pharm..

[36] Spomer N., Klingmann V., Stoltenberg I., Lerch C., Meissner T., Breitkreutz J. (2012). Acceptance of Uncoated Mini-Tablets in Young Children: Results from a Prospective Exploratory Cross-over Study. Arch. Dis. Child..

[37] Klingmann V., Linderskamp H., Meissner T., Mayatepek E., Moeltner A., Breitkreutz J., Bosse H.M. (2018). Acceptability of Multiple Uncoated Minitablets in Infants and Toddlers: A Randomized Controlled Trial. J. Pediatr..

[38] Batchelor H. (2019). Minitablets May Be an Acceptable Alternative to Liquid in Infants and Young Children. J. Pediatr..

[39] Wagner-Hattler L., Page S., Rode T. (2024). Minitablets Current Use and Future Opportunities—An APV Course on Manufacturing, Packaging, Characterization and Use of Minitablets. Eur. J. Pharm. Biopharm..

[40] Sznitowska M., Kluk A., Brandt A., Sznurkowska K., Plata-Nazar K., Mysliwiec M., Kaminska B., Kotlowska H. (2015). Can Preschool-Aged Children Swallow Several Minitablets at a Time? Results from a Clinical Pilot Study. Int. J. Pharm..

[41] Hejduk A., Lulek J. (2022). Dispensing of Minitablets—Has the Problem Been Resolved?. Int. J. Pharm..

[42] Pyteraf J., Jamróz W., Kurek M., Bąk U., Loskot J., Kramarczyk D., Paluch M., Jachowicz R. (2023). Preparation and Advanced Characterization of Highly Drug-Loaded, 3D Printed Orodispersible Tablets Containing Fluconazole. Int. J. Pharm..

[43] Tranová T., Loskot J., Navrátil O., Brniak W., Mužíková J. (2023). Effect of Co-Processed Excipient Type on Properties of Orodispersible Tablets Containing Captopril, Tramadol, and Domperidone. Int. J. Pharm..

[44] Obajtek N., Mendyk A., Szlęk J., Pacławski A. RKinetDS—Software for Modeling Dissolution Profiles. GitHub Repository. https://github.com/AleksanderMendyk/RKinetDS_deploy.

[45] Korsmeyer R.W., Gumy R., Doelker E., Buri P., Peppas N.A. (1983). Mechanisms of Solute Release from Porous Hydrophilic Polymers. Int. J. Pharm..

[46] Peppas N.A., Sahlin J.J. (1989). A Simple Equation for the Description of Solute Release. III. Coupling of Diffusion and Relaxation. Int. J. Pharm..

[47] Hixson A.W., Crowell J.H. (1931). Dependence of Reaction Velocity upon Surface and Agitation. Ind. Eng. Chem..

[48] Hopfenberg H.B., Paul D.R., Harris F.W. (1976). Controlled Release from Erodible Slabs, Cylinders, and Spheres. Controlled Release Polymeric Formulations.

[49] Costa P., Lobo J.M.S. (2001). Modeling and Comparison of Dissolution Profiles. Eur. J. Pharm. Sci..

[50] Bruschi M.L. (2015). Mathematical Models of Drug Release. Strategies to Modify the Drug Release from Pharmaceutical Systems.

[51] Podczeck F. (1993). Comparison of in Vitro Dissolution Profiles by Calculating Mean Dissolution Time (MDT) or Mean Residence Time (MRT). Int. J. Pharm..

[52] European Medicines Agency (EMA) (2010). Guideline on the Investigation of Bioequivalence.

[53] Detrich Á., Dömötör K.J., Katona M.T., Markovits I., Vargáné Láng J. (2019). Polymorphic Forms of Bisoprolol Fumarate: Preparation and Characterization. J. Therm. Anal. Calorim..

[54] Mahnaj T., Ahmed S.U., Plakogiannis F.M. (2013). Characterization of Ethyl Cellulose Polymer. Pharm. Dev. Technol..

[55] Davidovich-Pinhas M., Barbut S., Marangoni A.G. (2014). Physical Structure and Thermal Behavior of Ethylcellulose. Cellulose.

[56] U.S. Food and Drug Administration (2008). Guidance for Industry: Orally Disintegrating Tablets.

[57] Askarizadeh M., Esfandiari N., Honarvar B., Sajadian S.A., Azdarpour A. (2023). Kinetic Modeling to Explain the Release of Medicine from Drug Delivery Systems. ChemBioEng Rev..

[58] Wasilewska K., Szekalska M., Ciosek-Skibinska P., Lenik J., Basa A., Jacyna J., Markuszewski M., Winnicka K. (2019). Ethylcellulose in Organic Solution or Aqueous Dispersion Form in Designing Taste-Masked Microparticles by the Spray Drying Technique with a Model Bitter Drug: Rupatadine Fumarate. Polymers.

[59] Tran T.H., Ramasamy T., Poudel B.K., Marasini N., Moon B.K., Choo H.J., Choi H.-G., Yong C.S., Kim J.O. (2014). Preparation and Characterization of Spray-Dried Gelatin Microspheres Encapsulating Ganciclovir. Macromol. Res..

[60] Arpagaus C. (2019). PLA/PLGA Nanoparticles Prepared by Nano Spray Drying. J. Pharm. Investig..

[61] Goh H.P., Heng P.W.S., Liew C.V. (2018). Comparative Evaluation of Powder Flow Parameters with Reference to Particle Size and Shape. Int. J. Pharm..

[62] Wünsch I., Finke J.H., John E., Juhnke M., Kwade A. (2021). The Influence of Particle Size on the Application of Compression and Compaction Models for Tableting. Int. J. Pharm..

[63] Emeje M.O., Kunle O.O., Ofoefule S.I. (2006). Compaction Characteristics of Ethylcellulose in the Presence of Some Channeling Agents: Technical Note. AAPS PharmSciTech.

[64] Thoorens G., Krier F., Leclercq B., Carlin B., Evrard B. (2014). Microcrystalline Cellulose, a Direct Compression Binder in a Quality by Design Environment—A Review. Int. J. Pharm..

[65] Li J.Z., Rekhi G.S., Augsburger L.L., Shangraw R.F. (1996). The Role of Intra- and Extragranular Microcrystalline Cellulose in Tablet Dissolution. Pharm. Dev. Technol..

[66] Katikaneni P.R., Upadrashta S.M., Rowlings C.E., Neau S.H., Hileman G.A. (1995). Consolidation of Ethylcellulose: Effect of Particle Size, Press Speed, and Lubricants. Int. J. Pharm..

[67] Miller R.A., Leung E.M.K., Oates R.J. (1999). The Compression of Spheres Coated with an Aqueous Ethylcellulose Dispersion. Drug Dev. Ind. Pharm..

[68] Wasilewska K., Winnicka K. (2019). Ethylcellulose-a Pharmaceutical Excipient with Multidirectional Application in Drug Dosage Forms Development. Materials.

[69] García-Guzmán P., Schifter-Aceves L., Ortega-Almanza L., Romero-Canto P. (2024). Taguchi Design for Development of Lipid-Polymer Effervescent Floating Tablets for Metformin Prolonged Release. Int. J. Drug Deliv. Technol..

[70] Khan K.A., Ahmad A., Marini C., Nicotra M., Di Cerbo A., Fazal-Ur-Rehman, Ullah N., Khan G.M. (2024). Formulation and Preparation of Losartan-Potassium-Loaded Controlled-Release Matrices Using Ethocel Grade 10 to Establish a Correlation between In Vitro and In Vivo Results. Pharmaceutics.

[71] Al-Akayleh F., Alkhawaja B., Al-Zoubi N., Abdelmalek S.M.A., Daadoue S., AlAbbasi D., Al-Masri S., Ali Agha A.S.A., Rifai A., Olaimat A.R. (2025). Optimized Ethylcellulose Films Incorporating a Novel Clotrimazole Deep Eutectic System for Enhanced Permeation and Antifungal Activity. Int. J. Pharm..

