# Supplementary material for: Development of Prolonged-Release Orodispersible Minitablets Containing Bisoprolol Fumarate
Source: Pharmaceutics. 2026 Aug 27;18(9):1080. doi: 10.3390/pharmaceutics18091080 (PMC13610104; doi:10.3390/pharmaceutics18091080)
Supplement: Supplementary file 1 [file pharmaceutics-18-01080-s001.zip › pharmaceutics-4445471-supplementary.pdf]

Supplementary materials

Thermogravimetric analysis

Thermogravimetric analysis was performed to evaluate the mass-loss behavior of neat BF and EC as well as their spray-dried BF + EC systems (1:9, 1:1, and 9:1) upon heating. Measurements were carried out using a Mettler-Toledo TG 50 thermogravimetric analyzer coupled with a Mettler MT5 balance (Mettler-Toledo, Greifensee, Switzerland). Powder samples of approximately 10 mg were placed in open aluminum pans and heated under a nitrogen atmosphere with a purge flow rate of 50 mL/min at a heating rate of 10 K/min from 300 to 775 K.

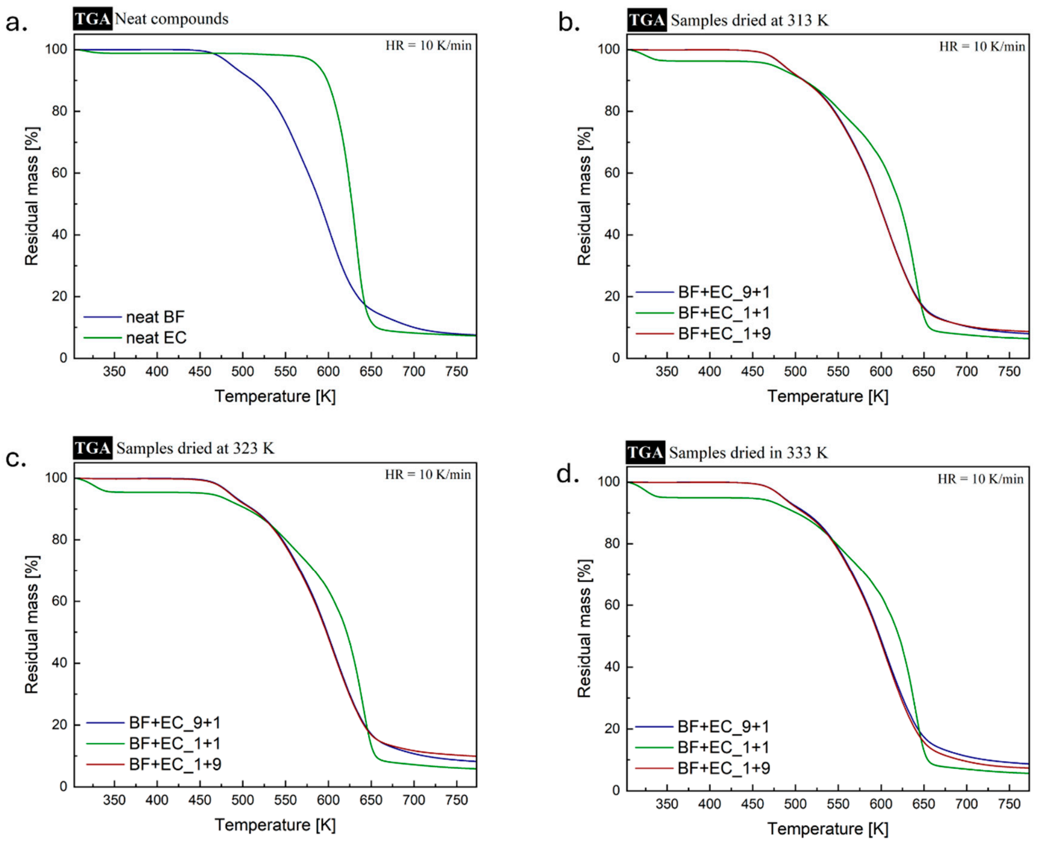

Figure S1: Thermogravimetric profiles of (a) neat BF and EC and the BF + EC 9:1, 1:1, and 1:9 formulations spray-dried at (b) 313 K, (c) 323 K, and (d) 333 K. The thermograms were used to assess the thermal behavior of the components and spray-dried microparticles, including the presence of low-temperature mass loss potentially attributable to residual solvent or moisture.

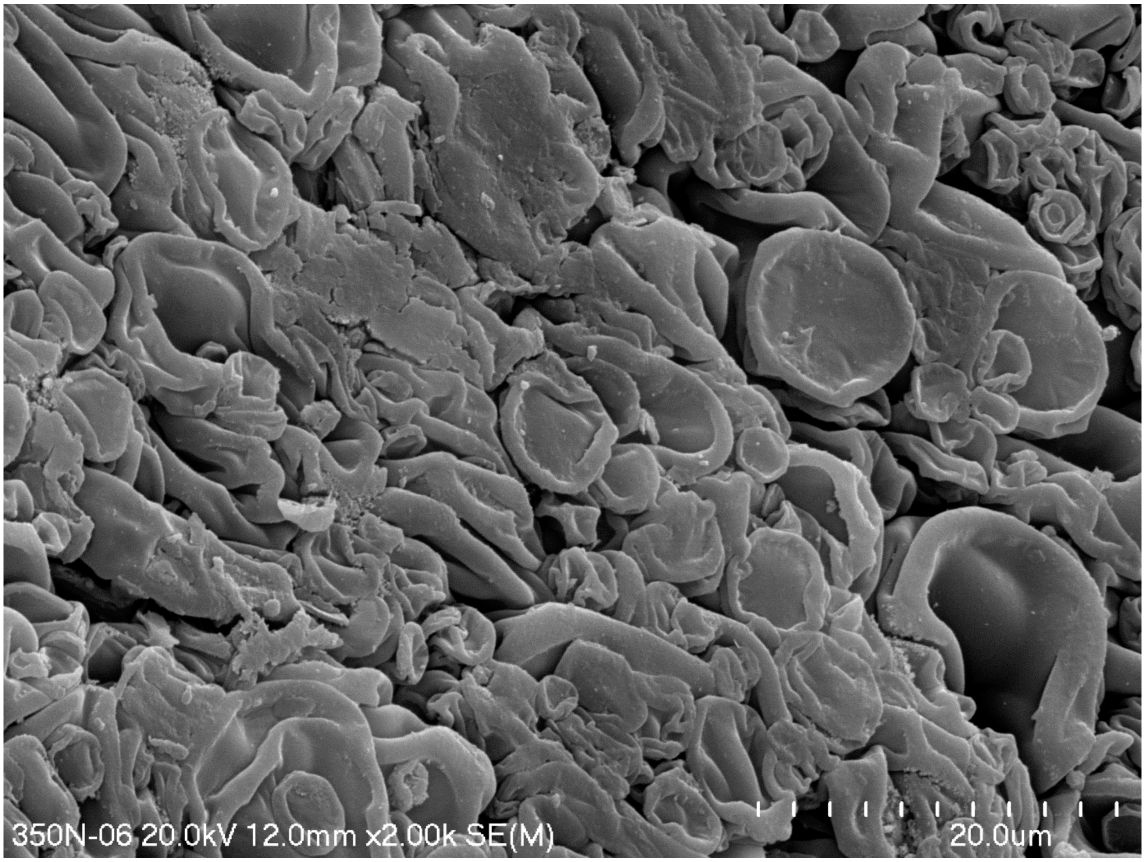

Figure S2: SEM image of lateral external surface of MT_BF_EC_1:9_350N at 2000× magnification, showing the external morphology of the minitablet after compression.

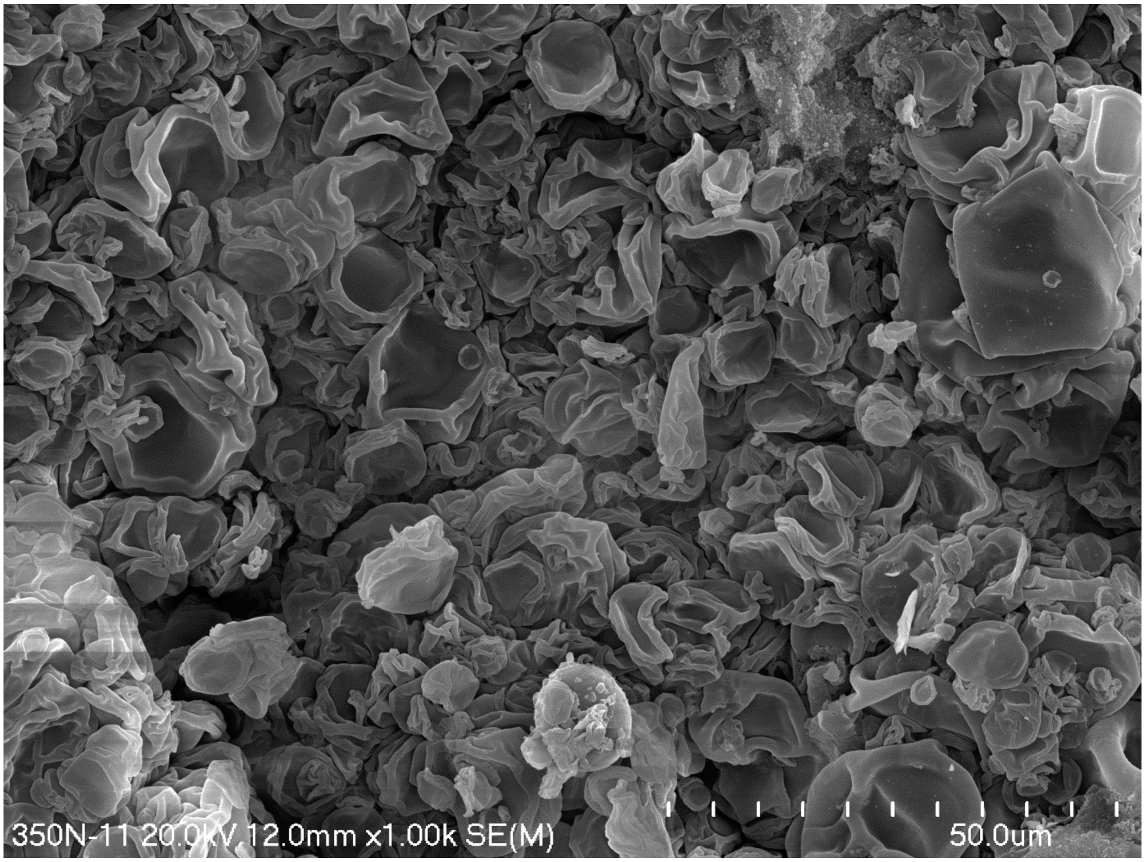

Figure S3: SEM image of fracture surface of MT_BF_EC_1:9_350N at 1000× magnification, showing the internal structure of the compressed minitablet and the distribution of microparticles within the tablet matrix.

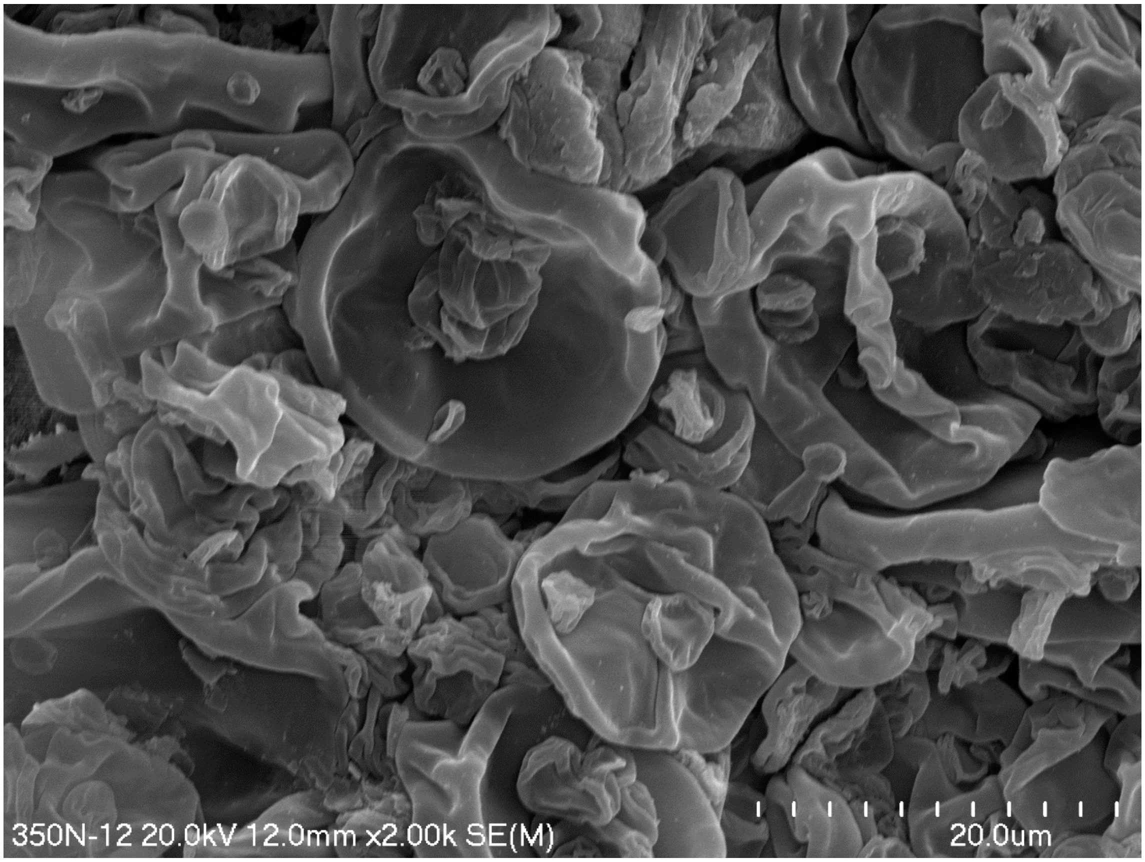

Figure S4: SEM image of fracture surface of MT_BF_EC_1:9_350N at 2000× magnification, providing a higher-magnification view of the internal structure of the compressed minitablet.

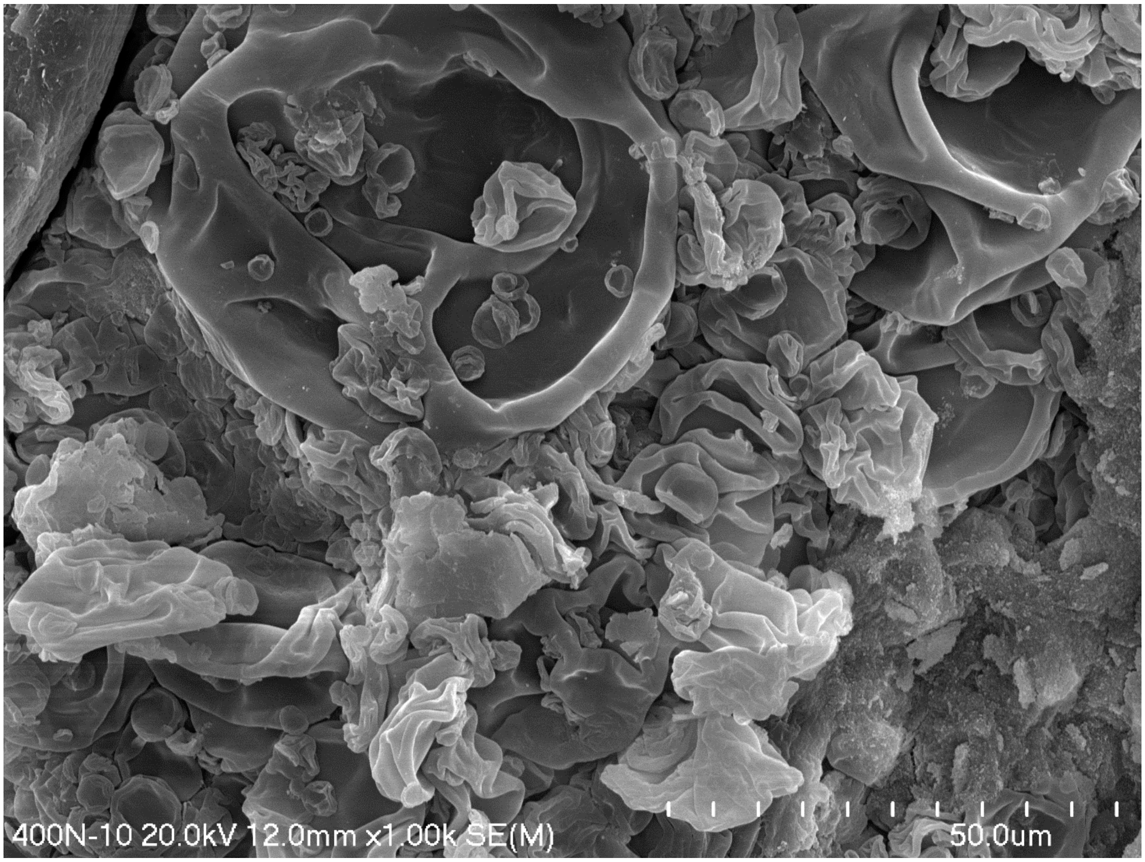

Figure S5: SEM image of fracture surface of MT_BF_EC_1:9_400N at 1000× magnification, showing the internal structure of the compressed minitablet and the distribution of microparticles within the tablet matrix.

Table S1. Properties of spray-dried microparticles: drug-loading parameters and process yield.

Formulation Name	Theoretical Drug Loading [%]	Actual Drug Loading [%]	Drug-Loading Efficiency [%]	Process Yield [%]	Drug Recovery [%]
BF_EC_9:1_40	90	88.09 ± 2.31	97.88 ± 2.57	51.8	50.7
BF_EC_9:1_50	90	87.17 ± 3.13	96.86 ± 3.48	44.3	42.91
BF_EC_9:1_60	90	88.17 ± 1.93	97.97 ± 2.14	65.5	64.17
BF_EC_3:1_40	75	74.51 ± 1.22	99.34 ± 1.63	50.1	49.77
BF_EC_3:1_50	75	74.33 ± 0.76	99.11 ± 1.02	61.8	61.25
BF_EC_3:1_60	75	76.43 ± 0.90	101.91 ± 1.20	66.8	68.08
BF_EC_1:1_40	50	52.70 ± 2.68	105.39 ± 5.35	49.7	52.38
BF_EC_1:1_50	50	54.15 ± 0.42	108.29 ± 0.84	59.2	64.11
BF_EC_1:1_60	50	55.23 ± 0.36	110.46 ± 0.71	56.5	62.41
BF_EC_1:3_40	25	25.54 ± 0.14	102.14 ± 0.54	38.9	39.73
BF_EC_1:3_50	25	25.34 ± 0.29	101.34 ± 1.15	36.5	36.99
BF_EC_1:3_60	25	25.26 ± 0.28	101.05 ± 1.14	45.0	45.47
BF_EC_1:9_40	10	9.84 ± 0.11	98.39 ± 1.07	22.9	22.53
BF_EC_1:9_50	10	9.40 ± 0.11	94.03 ± 1.09	36.7	34.51
BF_EC_1:9_60	10	9.51 ± 0.04	95.09 ± 0.44	49.5	47.07

Table S2. Statistical analysis parameters for particle size distribution: D_{50} values. The table presents the results of one-way ANOVA, including degrees of freedom (df), F-values, and p-values.

	DF	Sum of Squares	Mean Square	F-Value	Prob>F
**BF_EC_1:9—differences between temperatures**					
Model	2	4.11688	2.05844	163.29617	<0.0001
Error	15	0.18908	0.01261		
Total	17	4.30596			
**BF_EC_1:3—differences between temperatures**					
Model	2	32.13778	16.06889	3012.91667	<0.0001
Error	15	0.08	0.00533		
Total	17	32.21778			
**BF_EC_1:1—differences between temperatures**					
Model	2	37.92333	18.96167	2797.62295	<0.0001
Error	15	0.10167	0.00678		
Total	17	38.025			
**BF_EC_3:1—differences between temperatures**					
Model	2	9.60778	4.80389	882.34694	<0.0001
Error	15	0.08167	0.00544		
Total	17	9.68944			
**Differences between formulations spray-dried at 40°C**					
Model	3	139.94461	46.6482	9743.75022	<0.0001
Error	20	0.09575	0.00479		
Total	23	140.04036			
**Differences between formulations spray-dried at 50°C**					
Model	3	127.76833	42.58944	6552.22222	<0.0001
Error	20	0.13	0.0065		
Total	23	127.89833			
**Differences between formulations spray-dried at 60°C**					
Model	3	30.53833	10.17944	898.18627	<0.0001
Error	20	0.22667	0.01133		
Total	23	30.765			

Bold indicates significant differences ($p < 0.05$).

Table S3. Statistical analysis parameters for area under the dissolution curve (AUC) values calculated for microparticles. The table presents the results of one-way ANOVA, including degrees of freedom (df), F-values, and p-values.

	DF	Sum of Squares	Mean Square	F-Value	Prob>F
**BF_EC_1:9—differences between temperatures**					
Model	2	19.48553	9.74277	41.52657	3.05848E-4
Error	6	1.40769	0.23462		
Total	8	20.89323			
BF_EC_1:3—differences between temperatures					
Model	2	1.80512	0.90256	4.1833	0.07284
Error	6	1.29452	0.21575		
Total	8	3.09963			
**BF_EC_1:1—differences between temperatures**					
Model	2	4.83117	2.41559	35.81187	4.61817E-4
Error	6	0.40471	0.06745		
Total	8	5.23588			
**BF_EC_3:1—differences between temperatures**					
Model	2	0.16194	0.08097	6.9724	0.02722
Error	6	0.06968	0.01161		
Total	8	0.23161			
**BF_EC_9:1—differences between temperatures**					
Model	2	0.60704	0.30352	4.05736	0.07681
Error	6	0.44884	0.07481		
Total	8	1.05588			
**Differences between formulations spray-dried at 40°C**					
Model	4	131.41389	32.85347	213.18329	<0.0001
Error	10	1.54109	0.15411		
Total	14	132.95498			
**Differences between formulations spray-dried at 50°C**					
Model	4	46.87272	11.71818	106.22548	<0.0001
Error	10	1.10314	0.11031		
Total	14	47.97587			
**Differences between formulations spray-dried at 60°C**					
Model	4	146.58885	36.64721	373.49165	<0.0001
Error	10	0.98121	0.09812		
Total	14	147.57005			

Bold indicates significant differences ($p < 0.05$).

Table S4. Statistical analysis parameters for area under the dissolution curve (AUC) values calculated for minitablets. The table presents the results of one-way ANOVA, including degrees of freedom (df), F-values, and p-values.

	DF	Sum of Squares	Mean Square	F-Value	Prob>F
**MT_BF_EC_1:9—differences between compression forces and corresponding MP**					
Model	4	32.41748	8.10437	23.73791	4.33786E-5
Error	10	3.4141	0.34141		
Total	14	35.83158			
**MT_BF_EC_1:1—differences between compression forces and corresponding MP**					
Model	4	72.68297	18.17074	5.03847	0.01742
Error	10	36.06402	3.6064		
Total	14	108.74699			
**MT_BF_EC_9:1—differences between compression forces and corresponding MP**					
Model	4	44.44144	11.11036	1.44505	0.28937
Error	10	76.8854	7.68854		
Total	14	121.32684			
**Differences between MTs compressed with 150 N**					
Model	2	17.60583	8.80291	8.09517	0.01977
Error	6	6.52457	1.08743		
Total	8	24.1304			
**Differences between MTs compressed with 250 N**					
Model	2	34.05955	17.02977	9.60733	0.01347
Error	6	10.63549	1.77258		
Total	8	44.69504			
Differences between MTs compressed with 350 N					
Model	2	61.47444	30.73722	3.04643	0.12214
Error	6	60.53745	10.08957		
Total	8	122.01189			
Differences between MTs compressed with 400 N					
Model	2	26.73853	13.36926	2.09294	0.20439
Error	6	38.32671	6.38779		
Total	8	65.06524			

Bold indicates significant differences ($p < 0.05$).

Table S5. Statistical analysis parameters for mean dissolution time (MDT) values calculated for microparticles. The table presents the results of one-way ANOVA, including degrees of freedom (df), F-values, and p-values.

	DF	Sum of Squares	Mean Square	F-Value	Prob>F
**BF_EC_1:9—differences between temperatures**					
Model	2	19.48553	9.74277	41.52657	3.05848E-4
Error	6	1.40769	0.23462		
Total	8	20.89323			
BF_EC_1:3—differences between temperatures					
Model	2	1.80512	0.90256	4.1833	0.07284
Error	6	1.29452	0.21575		
Total	8	3.09963			
**BF_EC_1:1—differences between temperatures**					
Model	2	4.83117	2.41559	35.81187	4.61817E-4
Error	6	0.40471	0.06745		
Total	8	5.23588			
**BF_EC_3:1—differences between temperatures**					
Model	2	0.16194	0.08097	6.9724	0.02722
Error	6	0.06968	0.01161		
Total	8	0.23161			
BF_EC_9:1—differences between temperatures					
Model	2	0.60704	0.30352	4.05736	0.07681
Error	6	0.44884	0.07481		
Total	8	1.05588			
**Differences between formulations spray-dried at 40°C**					
Model	4	131.41389	32.85347	213.18329	<0.0001
Error	10	1.54109	0.15411		
Total	14	132.95498			
**Differences between formulations spray-dried at 50°C**					
Model	4	46.87272	11.71818	106.22548	<0.0001
Error	10	1.10314	0.11031		
Total	14	47.97587			
**Differences between formulations spray-dried at 60°C**					
Model	4	146.58885	36.64721	373.49165	<0.0001
Error	10	0.98121	0.09812		
Total	14	147.57005			

Bold indicates significant differences ($p < 0.05$).

Table S6. Statistical analysis parameters for mean dissolution time (MDT) values obtained for minitablets. The table presents the results of one-way ANOVA, including degrees of freedom (df), F-values, and p-values.

	DF	Sum of Squares	Mean Square	F-Value	Prob>F
**MT_BF_EC_1:9—differences between compression forces and corresponding MP**					
Model	4	4.4764	1.1191	25.54796	3.12482E-5
Error	10	0.43804	0.0438		
Total	14	4.91444			
MT_BF_EC_1:1—differences between compression forces and corresponding MP					
Model	4	0.14034	0.03509	0.90916	0.49483
Error	10	0.38591	0.03859		
Total	14	0.52626			
**MT_BF_EC_9:1—differences between compression forces and corresponding MP**					
Model	4	3.75147	0.93787	5.05064	0.01729
Error	10	1.85693	0.18569		
Total	14	5.60839			
**Differences between MTs compressed with 150 N**					
Model	2	14.92219	7.4611	646.23409	9.86642E-8
Error	6	0.06927	0.01155		
Total	8	14.99147			
**Differences between MTs compressed with 250 N**					
Model	2	16.40132	8.20066	135.64426	1.01311E-5
Error	6	0.36274	0.06046		
Total	8	16.76406			
**Differences between MTs compressed with 350 N**					
Model	2	5.00758	2.50379	10.32589	0.01141
Error	6	1.45486	0.24248		
Total	8	6.46245			
**Differences between MTs compressed with 400 N**					
Model	2	7.21303	3.60652	32.75293	5.90784E-4
Error	6	0.66068	0.11011		
Total	8	7.87371			

Bold indicates significant differences ($p < 0.05$).

Table S7. Results of Tukey's post hoc multiple-comparison test for area under the dissolution curve (AUC) values obtained for microparticles. The table presents pairwise comparisons between the investigated formulations, including 95% confidence intervals. Statistical significance was assumed at $\alpha = 0.05$.

Comparison	Mean Diff.	SEM	q-Value	Adj. p-Value	95% CI
**BF_EC_1:9—differences between temperatures**					
**BF_EC_1:9_40°C vs. BF_EC_1:9_50°C**	**2.69945**	**0.39549**	**9.65289**	**0.00118**	**(1.49; 3.91)**
BF_EC_1:9_60°C vs. BF_EC_1:9_40°C	-0.71849	0.39549	2.56923	0.24269	(-1.93; 0.50)
**BF_EC_1:9_50°C vs. BF_EC_1:9_60°C**	**-3.41794**	**0.39549**	**12.22212**	**3.25E-04**	**(-4.63; -2.20)**
**BF_EC_1:3—differences between temperatures**					
BF_EC_1:3_50°C vs. BF_EC_1:3_40°C	-0.90905	0.37926	3.38976	0.11642	(-2.07; 0.25)
BF_EC_1:3_60°C vs. BF_EC_1:3_40°C	0.07725	0.37926	0.28807	0.97747	(-1.09; 1.24)
BF_EC_1:3_60°C vs. BF_EC_1:3_50°C	0.9863	0.37926	3.67782	0.08982	(-0.18; 2.15)
**BF_EC_1:1—differences between temperatures**					
BF_EC_1:1_50°C vs. BF_EC_1:1_40°C	-0.57658	0.21206	3.84523	0.07731	(-1.23; 0.07)
**BF_EC_1:1_60°C vs. BF_EC_1:1_40°C**	**-1.76011**	**0.21206**	**11.73823**	**4.07E-04**	**(-2.41; 1.11)**
**BF_EC_1:1_60°C vs. BF_EC_1:1_50°C**	**-1.18353**	**0.21206**	**7.893**	**0.00339**	**(-1.83; -0.53)**
**BF_EC_3:1—differences between temperatures**					
BF_EC_3:1_50°C vs. BF_EC_3:1_40°C	0.1861	0.08799	2.9912	0.16677	(-0.08; 0.46)
BF_EC_3:1_60°C vs. BF_EC_3:1_40°C	-0.14146	0.08799	2.27358	0.31307	(-0.41; 0.13)
**BF_EC_3:1_60°C vs. BF_EC_3:1_50°C**	**-0.32756**	**0.08799**	**5.26478**	**0.02291**	**(-0.60; 0.06)**
**BF_EC_9:1—differences between temperatures**					
BF_EC_9:1_50°C vs. BF_EC_9:1_40°C	0.2811	0.22332	1.78011	0.46563	(-0.40; 0.97)
BF_EC_9:1_60°C vs. BF_EC_9:1_40°C	0.63477	0.22332	4.01983	0.06619	(-0.05; 1.32)
BF_EC_9:1_60°C vs. BF_EC_9:1_50°C	0.35367	0.22332	2.23972	3.22E-01	(-0.33; 1.04)
**Differences between formulations spray-dried at 40°C**					
**BF:EC_1:3 vs. BF:EC_9:1**	**7.46424**	**0.32053**	**32.93306**	**0**	**(-6.41; 8.52)**
**BF:EC_1:1 vs. BF:EC_9:1**	**8.14066**	**0.32053**	**35.91751**	**0**	**(7.09; 9.20)**
BF:EC_1:1 vs. BF:EC_1:3	0.67642	0.32053	2.98445	0.28716	(-0.38; 1.73)
**BF:EC_3:1 vs. BF:EC_9:1**	**6.50593**	**0.32053**	**28.7049**	**0**	**(5.45; 7.56)**
BF:EC_3:1 vs. BF:EC_1:3	-0.95831	0.32053	4.22816	0.07967	(-2.01; 0.10)
**BF:EC_3:1 vs. BF:EC_1:1**	**-1.63473**	**0.32053**	**7.21261**	**0.00329**	**(-2.96; -0.58)**
**BF:EC_1:9 vs. BF:EC_9:1**	**6.99164**	**0.32053**	**30.84791**	**0**	**(5.94; 8.05)**
BF:EC_9:1 vs. BF:EC_1:3	-0.4726	0.32053	2.08515	0.59901	(-1.53; 0.58)
**BF:EC_9:1 vs. BF:EC_1:1**	**-1.14902**	**0.32053**	**5.0696**	**0.03169**	**(-2.20; -0.09)**
BF:EC_9:1 vs. BF:EC_3:1	0.48571	0.32053	2.14301	0.57595	(-0.57; 1.54)
**Differences between formulations spray-dried at 50°C**					
BF:EC_3:1 vs. BF:EC_9:1	-0.58071	0.27119	3.02831	0.27551	(-1.47; 0.31)
BF:EC_1:1 vs. BF:EC_9:1	0.29134	0.27119	1.51932	0.81544	(-0.60; 1.18)
BF:EC_1:1 vs. BF:EC_3:1	0.87205	0.27119	4.54763	0.05621	(-0.02; 1.76)
BF:EC_1:3 vs. BF:EC_9:1	-0.71755	0.27119	3.74192	0.13419	(-1.61; 0.17)
BF:EC_1:3 vs. BF:EC_3:1	-0.13684	0.27119	0.71361	0.98501	(-1.03; 0.76)
**BF:EC_1:3 vs. BF:EC_1:1**	**-1.00889**	**0.27119**	**5.26125**	**0.02569**	**(-1.90; -0.12)**
**BF:EC_1:9 vs. BF:EC_9:1**	**-4.57329**	**0.27119**	**23.8492**	**0**	**(-5.47; -3.68)**
**BF:EC_1:9 vs. BF:EC_3:1**	**-3.99259**	**0.27119**	**20.82089**	**3.38E-07**	**(-4.89; -3.10)**

**BF:EC_1:9 vs. BF:EC_1:1**	**-4.86463**	**0.27119**	**25.36852**	**0**	**(-5.76; -3.97)**
**BF:EC_1:9 vs. BF:EC_1:3**	**-3.85575**	**0.27119**	**20.10727**	**4.67E-07**	**(-4.75; -2.96)**
**Differences between formulations spray-dried at 60°C**					
**BF:EC_1:3 vs. BF:EC_9:1**	**8.25998**	**0.25576**	**45.67301**	**0**	**(7.42; 9.10)**
**BF:EC_1:9 vs. BF:EC_9:1**	**8.3449**	**0.25576**	**46.14258**	**0**	**(7.50; 9.19)**
BF:EC_9:1 vs. BF:EC_1:3	0.08492	0.25576	0.46957	0.99692	(-0.76; 0.93)
**BF:EC_1:1 vs. BF:EC_1:9**	**7.09904**	**0.25576**	**39.25367**	**0**	**(6.26; 7.94)**
**BF:EC_1:1 vs. BF:EC_1:3**	**-1.16094**	**0.25576**	**6.41934**	**0.00742**	**(-2.00; -0.32)**
**BF:EC_1:1 vs. BF:EC_9:1**	**-1.24586**	**0.25576**	**6.88891**	**0.00457**	**(-2.09; -0.40)**
**BF:EC_3:1 vs. BF:EC_1:9**	**7.08297**	**0.25576**	**39.16478**	**0**	**(6.24; 7.92)**
**BF:EC_3:1 vs. BF:EC_1:3**	**-1.17701**	**0.25576**	**6.50823**	**0.00676**	**(-2.02; -0.34)**
**BF:EC_3:1 vs. BF:EC_9:1**	**-1.26194**	**0.25576**	**6.9778**	**0.00417**	**(-2.10; -0.42)**
BF:EC_3:1 vs. BF:EC_1:1	-0.01608	0.25576	0.08889	1	(-0.86; 0.83)

Bold indicates significant differences ($p < 0.05$).

Mean Diff.—mean difference, **SEM**—standard error of the mean difference, **CI**—confidence interval, and **Adj. p-Value**—adjusted p-value.

Table S8. Results of Tukey's post hoc multiple-comparison test for area under the dissolution curve (AUC) values obtained for minitablets. The table presents pairwise comparisons between the investigated formulations, including 95% confidence intervals. Statistical significance was assumed at $\alpha = 0.05$.

Comparison	Mean Diff.	SEM	q-Value	Adj. p-Value	95% CI
**MT_BF_EC_1:9—differences between compression forces and corresponding MP**					
MT_BF_EC_1:9_250 vs. MT_BF_EC_1:9_150	-0.88321	0.47708	2.6181	0.39886	(-2.45; 0.69)
**MT_BF_EC_1:9_350 vs. MT_BF_EC_1:9_150**	**-4.03296**	**0.47708**	**11.95492**	**5.52E-05**	**(-5.60; -2.46)**
**MT_BF_EC_1:9_350 vs. MT_BF_EC_1:9_250**	**-3.14975**	**0.47708**	**9.33681**	**4.50E-04**	**(-4.72; -1.58)**
**MT_BF_EC_1:9_400 vs. MT_BF_EC_1:9_150**	**-3.19264**	**0.47708**	**9.46396**	**4.03E-04**	**(-4.76; -1.62)**
**MT_BF_EC_1:9_400 vs. MT_BF_EC_1:9_250**	**-2.30943**	**0.47708**	**6.84585**	**0.00477**	**(-3.88; -0.74)**
MT_BF_EC_1:9_400 vs. MT_BF_EC_1:9_350	0.84032	0.47708	2.49096	0.44327	(-0.73; 2.41)
**BF_EC_1:9_60°C vs. MT_BF_EC_1:9_150**	**-2.11545**	**0.47708**	**6.27082**	**0.00867**	**(-3.69; -0.55)**
BF_EC_1:9_60°C vs. MT_BF_EC_1:9_250	-1.23224	0.47708	3.65272	0.14736	(-2.80; 0.34)
**BF_EC_1:9_60°C vs. MT_BF_EC_1:9_350**	**1.91752**	**0.47708**	**5.68409**	**0.01623**	**(0.35; 3.49)**
BF_EC_1:9_60°C vs. MT_BF_EC_1:9_400	1.0772	0.47708	3.19313	0.23498	(-0.49; 2.65)
**MT_BF_EC_1:1—differences between compression forces and corresponding MP**					
MT_BF_EC_1:1 _150 vs. BF_EC_1:1_60°C	-3.62028	1.55057	3.30191	0.211	(-8.72; 1.48)
MT_BF_EC_1:1 _250 vs. BF_EC_1:1_60°C	-2.58276	1.55057	2.35563	0.49325	(-7.69; 2.52)
MT_BF_EC_1:1 _250 vs. MT_BF_EC_1:1 _150	1.03752	1.55057	0.94628	0.95879	(-4.07; 6.14)
**MT_BF_EC_1:1 _350 vs. BF_EC_1:1_60°C**	**-5.58376**	**1.55057**	**5.09273**	**0.0309**	**(-10.69; -0.48)**
MT_BF_EC_1:1 _350 vs. MT_BF_EC_1:1 _150	-1.96348	1.55057	1.79082	0.71591	(-7.07; 3.14)
MT_BF_EC_1:1 _350 vs. MT_BF_EC_1:1 _250	-3.001	1.55057	2.7371	0.35982	(-8.10; 2.10)
**MT_BF_EC_1:1 _400 vs. BF_EC_1:1_60°C**	**-6.10984**	**1.55057**	**5.57254**	**0.01831**	**(-11.21; -1.01)**
MT_BF_EC_1:1 _400 vs. MT_BF_EC_1:1 _150	-2.48956	1.55057	2.27063	0.52582	(-7.59; 2.61)
MT_BF_EC_1:1 _400 vs. MT_BF_EC_1:1 _250	-3.52708	1.55057	3.21691	0.22955	(-8.63; 1.58)
MT_BF_EC_1:1 _400 vs. MT_BF_EC_1:1 _350	-0.52608	1.55057	0.47981	0.99666	(-5.63; 4.58)
**MT_BF_EC_9:1—differences between compression forces and corresponding MP**					
MT_BF_EC_9:1_150 vs. BF_EC_9:1_60°C	-2.82586	2.264	1.76518	0.72581	(-10.28; 4.63)
MT_BF_EC_9:1_250 vs. BF_EC_9:1_60°C	-2.48048	2.264	1.54944	0.80511	(-9.93; 4.97)
MT_BF_EC_9:1_250 vs. MT_BF_EC_9:1_150	0.34538	2.264	0.21574	0.99985	(-7.11; 7.80)
MT_BF_EC_9:1_350 vs. BF_EC_9:1_60°C	-3.86637	2.264	2.41514	0.47096	(-11.32; 3.58)
MT_BF_EC_9:1_350 vs. MT_BF_EC_9:1_150	-1.04051	2.264	0.64996	0.9894	(-8.49; 6.41)
MT_BF_EC_9:1_350 vs. MT_BF_EC_9:1_250	-1.38588	2.264	0.8657	0.96984	(-8.84; 6.07)
MT_BF_EC_9:1_400 vs. BF_EC_9:1_60°C	-5.20036	2.264	3.24842	0.22252	(-12.65; 2.25)
MT_BF_EC_9:1_400 vs. MT_BF_EC_9:1_150	-2.3745	2.264	1.48324	0.82751	(-9.83; 5.08)
MT_BF_EC_9:1_400 vs. MT_BF_EC_9:1_250	-2.71988	2.264	1.69898	0.751	(-10.17; 4.73)
MT_BF_EC_9:1_400 vs. MT_BF_EC_9:1_350	-1.334	2.264	0.83328	0.97367	(-8.79; 6.12)
**Differences between MTs compressed with 150 N**					
MT_BF_EC_1:1 _150 vs. MT_BF_EC_1:9_150	1.36332	0.85144	2.26442	0.3155	(-1.29; 3.98)
**MT_BF_EC_9:1_150 vs. MT_BF_EC_1:9_150**	**3.4036**	**0.85144**	**5.65325**	**0.01678**	**(0.79; 6.02)**
MT_BF_EC_9:1_150 vs. MT_BF_EC_1:1 _150	2.04028	0.85144	3.38883	0.11652	(-1.99; 4.65)
**Differences between MTs compressed with 250 N**					
MT_BF_EC_1:1 _250 vs. MT_BF_EC_1:9_250	3.28405	1.08707	4.27235	0.053	(-0.05; 6.62)
**MT_BF_EC_9:1_250 vs. MT_BF_EC_1:9_250**	**4.63218**	**1.08707**	**6.02619**	**0.01257**	**(1.30; 7.97)**
MT_BF_EC_9:1_250 vs. MT_BF_EC_1:1 _250	1.34814	1.08707	1.75384	0.47493	(-1.99; 4.68)

**Differences between MTs compressed with 350 N**					
MT_BF_EC_1:1 _350 vs. MT_BF_EC_1:9_350	3.4328	2.59353	1.87185	0.43401	(-4.53; 11.39)
MT_BF_EC_9:1_350 vs. MT_BF_EC_1:9_350	6.39605	2.59353	3.48768	0.10658	(-1.56; 14.35)
MT_BF_EC_9:1_350 vs. MT_BF_EC_1:1 _350	2.96326	2.59353	1.61582	0.52549	(-4.99; 10.92)
**Differences between MTs compressed with 400 N**					
MT_BF_EC_1:1 _400 vs. MT_BF_EC_1:9_400	2.0664	2.06362	1.41612	0.60273	(-4.27; 8.40)
MT_BF_EC_9:1_400 vs. MT_BF_EC_1:9_400	4.22174	2.06362	2.89319	0.1821	(-2.11; 10.55)
MT_BF_EC_9:1_400 vs. MT_BF_EC_1:1 _400	2.15534	2.06362	1.47707	0.57875	(-4.18; 8.49)

Bold indicates significant differences ($p < 0.05$).

Mean Diff.—mean difference, **SEM**—standard error of the mean difference, **CI**—confidence interval, and **Adj. p-Value**—adjusted p-value.

Table S9. Results of Tukey's post hoc multiple-comparison test for mean dissolution time (MDT) values obtained for microparticles. The table presents pairwise comparisons between the investigated formulations, including 95% confidence intervals. Statistical significance was assumed at $\alpha = 0.05$.

Comparison	Mean Diff.	SEM	q-Value	Adj. p-Value	95% CI
**BF_EC_1:9—differences between temperatures**					
**BF_EC_1:9_40°C vs. BF_EC_1:9_50°C**	1.1224	0.16656	9.52993	0.00127	(0.61; 1.63)
**BF_EC_1:9_60°C vs. BF_EC_1:9_50°C**	1.17417	0.16656	9.96954	0.000994518	(0.66; 1.69)
BF_EC_1:9_40°C vs. BF_EC_1:9_60°C	0.05178	0.16656	0.43961	0.94858	(-0.46; 0.56)
**BF_EC_1:3—differences between temperatures**					
BF_EC_1:3_50°C vs. BF_EC_1:3_40°C	-0.03851	0.03838	1.4187	0.6017	(-0.16; 0.08)
**BF_EC_1:3_60°C vs. BF_EC_1:3_40°C**	-0.19794	0.03838	7.29282	0.00505	(-0.32; -0.08)
**BF_EC_1:3_60°C vs. BF_EC_1:3_50°C**	-0.15943	0.03838	5.87411	0.01413	(-0.28; -0.04)
**BF_EC_1:1—differences between temperatures**					
BF_EC_1:1_50°C vs. BF_EC_1:1_40°C	-0.05375	0.03807	1.99678	0.39327	(-0.17; 0.06)
**BF_EC_1:1_60°C vs. BF_EC_1:1_40°C**	**0.35014**	**0.03807**	**13.00792**	**2.29E-04**	**(0.23; 0.47)**
**BF_EC_1:1_60°C vs. BF_EC_1:1_50°C**	**0.40389**	**0.03807**	**15.0047**	**1.02E-04**	**(0.29; 0.52)**
**BF_EC_3:1—differences between temperatures**					
**BF_EC_3:1_50°C vs. BF_EC_3:1_40°C**	**0.25043**	**0.0514**	**6.89065**	**0.00667**	**(0.09; 0.41)**
**BF_EC_3:1_60°C vs. BF_EC_3:1_40°C**	**0.38391**	**0.0514**	**10.56339**	**7.27E-04**	**(0.23; 0.54)**
BF_EC_3:1_60°C vs. BF_EC_3:1_50°C	0.13348	0.0514	3.67274	0.09023	(-0.02; 0.29)
**BF_EC_9:1—differences between temperatures**					
**BF_EC_9:1_50°C vs. BF_EC_9:1_40°C**	**0.21482**	**0.05333**	**5.6972**	**0.01621**	**(0.05; 0.38)**
**BF_EC_9:1_60°C vs. BF_EC_9:1_40°C**	**0.40296**	**0.05333**	**10.68673**	**6.82E-04**	**(0.24; 0.57)**
**BF_EC_9:1_60°C vs. BF_EC_9:1_50°C**	**0.18814**	**0.05333**	**4.98953**	**0.02873**	**(0.02; 0.35)**
**Differences between formulations spray-dried at 40°C**					
BF:EC_3:1 vs. BF:EC_9:1	0.02433	0.0865	0.39787	0.99838	(-0.26; 0.31)
BF:EC_1:1 vs. BF:EC_9:1	0.22477	0.0865	3.67493	0.14398	(-0.06; 0.51)
BF:EC_1:1 vs. BF:EC_3:1	0.20043	0.0865	3.27706	0.21629	(-0.08; 0.49)
**BF:EC_1:3 vs. BF:EC_9:1**	**0.74229**	**0.0865**	**12.13634**	**4.83E-05**	**(0.46; 1.03)**
**BF:EC_1:3 vs. BF:EC_3:1**	**0.71796**	**0.0865**	**11.73847**	**6.48E-05**	**(0.43; 1.00)**
**BF:EC_1:3 vs. BF:EC_1:1**	**0.51752**	**0.0865**	**8.46141**	**9.89E-04**	**(0.23; 0.80)**
**BF:EC_1:9 vs. BF:EC_9:1**	**3.11288**	**0.0865**	**50.89514**	**0**	**(2.83; 3.40)**
**BF:EC_1:9 vs. BF:EC_3:1**	**3.08855**	**0.0865**	**50.49727**	**0**	**(2.80; 3.37)**
**BF:EC_1:9 vs. BF:EC_1:1**	**2.88811**	**0.0865**	**47.22021**	**0**	**(2.60; 3.17)**
**BF:EC_1:9 vs. BF:EC_1:3**	**2.37059**	**0.0865**	**38.7588**	**0**	**(2.09; 2.66)**
**Differences between formulations spray-dried at 50°C**					
BF:EC_3:1 vs. BF:EC_9:1	0.05994	0.05682	1.4918	0.82468	(-0.13; 0.25)
BF:EC_1:1 vs. BF:EC_9:1	-0.0438	0.05682	1.09017	0.9334	(-0.23; 0.14)
BF:EC_1:1 vs. BF:EC_3:1	-0.10375	0.05682	2.58197	0.41121	(-0.29; 0.08)
**BF:EC_1:3 vs. BF:EC_9:1**	**0.48896**	**0.05682**	**12.16889**	**4.72E-05**	**(0.30; 0.68)**
**BF:EC_1:3 vs. BF:EC_3:1**	**0.42902**	**0.05682**	**10.67709**	**1.47E-04**	**(0.24; 0.62)**
**BF:EC_1:3 vs. BF:EC_1:1**	**0.53276**	**0.05682**	**13.25906**	**2.20E-05**	**(0.35; 0.72)**
**BF:EC_1:9 vs. BF:EC_9:1**	**1.77566**	**0.05682**	**44.19138**	**0**	**(1.59; 1.96)**
**BF:EC_1:9 vs. BF:EC_3:1**	**1.71572**	**0.05682**	**42.69958**	**0**	**(1.53; 1.90)**

**BF:EC_1:9 vs. BF:EC_1:1**	**1.81946**	**0.05682**	**45.28155**	**0**	**(1.63; 2.01)**
**BF:EC_1:9 vs. BF:EC_1:3**	**1.2867**	**0.05682**	**32.02249**	**0**	**(1.10; 1.47)**
**Differences between formulations spray-dried at 60°C**					
BF:EC_3:1 vs. BF:EC_9:1	0.00528	0.10478	0.0713	1	(-0.34; 0.35)
BF:EC_1:1 vs. BF:EC_9:1	0.17194	0.10478	2.32065	0.50655	(-0.17; 0.52)
BF:EC_1:1 vs. BF:EC_3:1	0.16666	0.10478	2.24935	0.53409	(-0.18; 0.51)
BF:EC_1:3 vs. BF:EC_9:1	0.14139	0.10478	1.90824	0.66972	(-0.20; 0.49)
BF:EC_1:3 vs. BF:EC_3:1	0.1361	0.10478	1.83694	0.6979	(-0.21; 0.48)
BF:EC_1:3 vs. BF:EC_1:1	-0.03056	0.10478	0.4124	0.99814	(-0.38; 0.31)
**BF:EC_1:9 vs. BF:EC_9:1**	**2.76169**	**0.10478**	**37.27372**	**0**	**(2.42; 3.11)**
**BF:EC_1:9 vs. BF:EC_3:1**	**2.75641**	**0.10478**	**37.20241**	**0**	**(2.41; 3.10)**
**BF:EC_1:9 vs. BF:EC_1:1**	**2.58975**	**0.10478**	**34.95307**	**0**	**(2.42; 2.93)**
**BF:EC_1:9 vs. BF:EC_1:3**	**2.6203**	**0.10478**	**35.36547**	**0**	**(2.28; 2.97)**

Bold indicates significant differences ($p < 0.05$).

Mean Diff.—mean difference, **SEM**—standard error of the mean difference, **CI**—confidence interval, and **Adj. p-Value**—adjusted p-value.

Table S10. Results of Tukey's post hoc multiple-comparison test for mean dissolution time (MDT) values obtained for minitablets. The table presents pairwise comparisons between the investigated formulations, including 95% confidence intervals. Statistical significance was assumed at $\alpha = 0.05$.

Comparison	Mean Diff.	SEM	q-Value	Adj. p-Value	95% CI
**MT_BF_EC_1:9—differences between compression forces and corresponding MP**					
MT_BF_EC_1:9_250 vs. MT_BF_EC_1:9_150	0.14181	0.17089	1.17356	0.91528	(-0.42; 0.70)
**MT_BF_EC_1:9_350 vs. MT_BF_EC_1:9_150**	**-1.29057**	**0.17089**	**10.6804**	**0.000147**	**(-1.85; -0.73)**
**MT_BF_EC_1:9_350 vs. MT_BF_EC_1:9_250**	**-1.43238**	**0.17089**	**11.85396**	**0.000059**	**(-1.99; 0.87)**
**MT_BF_EC_1:9_400 vs. MT_BF_EC_1:9_150**	**-0.95568**	**0.17089**	**7.90894**	**0.00166**	**(-1.52; -0.39)**
**MT_BF_EC_1:9_400 vs. MT_BF_EC_1:9_250**	**-1.09749**	**0.17089**	**9.0825**	**0.00056**	**(-1.66; -0.53)**
MT_BF_EC_1:9_400 vs. MT_BF_EC_1:9_350	0.33489	0.17089	2.77146	0.34903	(-0.22; 0.90)
BF_EC_1:9_60°C vs. MT_BF_EC_1:9_150	-0.54507	0.17089	4.51085	0.05852	(-1.11; 0.02)
**BF_EC_1:9_60°C vs. MT_BF_EC_1:9_250**	**-0.68688**	**0.17089**	**5.68441**	**0.01622**	**(-1.25; -0.12)**
**BF_EC_1:9_60°C vs. MT_BF_EC_1:9_350**	**0.7455**	**0.17089**	**6.16955**	**0.00965**	**(0.18; 1.31)**
BF_EC_1:9_60°C vs. MT_BF_EC_1:9_400	0.41061	0.17089	3.39809	0.19154	(-0.15; 0.97)
**MT_BF_EC_1:1—differences between compression forces and corresponding MP**					
MT_BF_EC_1:1 _150 vs. BF_EC_1:1_60°C	0.22172	0.1604	1.95492	0.65111	(-0.31; 0.75)
MT_BF_EC_1:1 _250 vs. BF_EC_1:1_60°C	0.18633	0.1604	1.64289	0.7718	(-0.34; 0.71)
MT_BF_EC_1:1 _250 vs. MT_BF_EC_1:1 _150	-0.03539	0.1604	0.31203	0.99938	(-0.56; 0.49)
MT_BF_EC_1:1 _350 vs. BF_EC_1:1_60°C	0.01712	0.1604	0.15095	0.99996	(-0.51; 0.55)
MT_BF_EC_1:1 _350 vs. MT_BF_EC_1:1 _150	-0.2046	0.1604	1.80397	0.71079	(-0.73; 0.32)
MT_BF_EC_1:1 _350 vs. MT_BF_EC_1:1 _250	-0.16921	0.1604	1.49194	0.82463	(-0.70; 0.36)
MT_BF_EC_1:1 _400 vs. BF_EC_1:1_60°C	0.00755	0.1604	0.06659	1	(-0.52; 0.54)
MT_BF_EC_1:1 _400 vs. MT_BF_EC_1:1 _150	-0.21417	0.1604	1.88833	0.67762	(-0.74; 0.31)
MT_BF_EC_1:1 _400 vs. MT_BF_EC_1:1 _250	-0.17878	0.1604	1.5763	0.79573	(-0.71; 0.35)
MT_BF_EC_1:1 _400 vs. MT_BF_EC_1:1 _350	-0.00957	0.1604	0.08436	1	(-0.54; 0.52)
**MT_BF_EC_9:1—differences between compression forces and corresponding MP**					
MT_BF_EC_9:1_150 vs. BF_EC_9:1_60°C	0.80308	0.35185	3.2279	0.22708	(-0.35; 1.96)
MT_BF_EC_9:1_250 vs. BF_EC_9:1_60°C	0.88449	0.35185	3.55515	0.16309	(-0.27; 2.04)
MT_BF_EC_9:1_250 vs. MT_BF_EC_9:1_150	0.08142	0.35185	0.32725	0.99925	(-1.08; 1.24)
MT_BF_EC_9:1_350 vs. BF_EC_9:1_60°C	1.10337	0.35185	4.43489	0.06359	(-0.05; 2.26)
MT_BF_EC_9:1_350 vs. MT_BF_EC_9:1_150	0.30029	0.35185	1.20699	0.90733	(-0.86; 1.46)
MT_BF_EC_9:1_350 vs. MT_BF_EC_9:1_250	0.21887	0.35185	0.87974	0.96807	(-0.94; 1.38)
**MT_BF_EC_9:1_400 vs. BF_EC_9:1_60°C**	**1.52926**	**0.35185**	**6.14672**	**0.00989**	**(0.37; 2.69)**
MT_BF_EC_9:1_400 vs. MT_BF_EC_9:1_150	0.72618	0.35185	2.91882	0.3053	(-0.43; 1.88)
MT_BF_EC_9:1_400 vs. MT_BF_EC_9:1_250	0.64476	0.35185	2.59157	0.40791	(-0.51; 1.80)
MT_BF_EC_9:1_400 vs. MT_BF_EC_9:1_350	0.42589	0.35185	1.71183	0.74616	(-0.73; 1.58)
**Differences between MTs compressed with 150 N**					
**MT_BF_EC_1:1 _150 vs. MT_BF_EC_1:9_150**	**-2.9131**	**0.08773**	**46.95795**	**7.36E-08**	**(-3.18; -2.64)**
**MT_BF_EC_9:1_150 vs. MT_BF_EC_1:9_150**	**-2.50369**	**0.08773**	**40.35842**	**2.57E-07**	**(-2.77; -2.23)**
**MT_BF_EC_9:1_150 vs. MT_BF_EC_1:1 _150**	**0.40941**	**0.08773**	**6.59953**	**0.00821**	**(0.14; 0.68)**
**Differences between MTs compressed with 250 N**					
**MT_BF_EC_1:1 _250 vs. MT_BF_EC_1:9_250**	**-3.09029**	**0.20076**	**21.76892**	**1.17E-05**	**(-3.71; -2.47)**
MT_BF_EC_9:1_250 vs. MT_BF_EC_1:9_250	-2.56408	0.20076	18.06209	3.49E-05	(-3.18; -1.95)
MT_BF_EC_9:1_250 vs. MT_BF_EC_1:1 _250	0.52622	0.20076	3.70683	0.08751	(-0.09; 1.14)

**Differences between MTs compressed with 350 N**					
**MT_BF_EC_1:1 _350 vs. MT_BF_EC_1:9_350**	**-1.82713**	**0.40206**	**6.42678**	**0.00931**	**(-3.06; -0.59)**
MT_BF_EC_9:1_350 vs. MT_BF_EC_1:9_350	-0.91282	0.40206	3.21079	0.13684	(-2.15; 0.32)
MT_BF_EC_9:1_350 vs. MT_BF_EC_1:1 _350	0.9143	0.40206	3.216	0.13619	(-0.32; 2.15)
**Differences between MTs compressed with 400 N**					
**MT_BF_EC_1:1 _400 vs. MT_BF_EC_1:9_400**	**-2.17158**	**0.27094**	**11.33492**	**4.93E-04**	**(-3.00; -1.34)**
MT_BF_EC_9:1_400 vs. MT_BF_EC_1:9_400	-0.82182	0.27094	4.28964	0.05221	(-1.65; 0.01)
**MT_BF_EC_9:1_400 vs. MT_BF_EC_1:1 _400**	**1.34976**	**0.27094**	**7.04529**	**0.00598**	**(0.52; 2.18)**

Bold indicates significant differences ($p < 0.05$).

Mean Diff.—mean difference, **SEM**—standard error of the mean difference, **CI**—confidence interval, and **Adj. p-Value**—adjusted p-value.
